# Metabolomic profiling reveals early biomarkers of gestational diabetes mellitus and associated hepatic steatosis

**DOI:** 10.1186/s12933-025-02645-4

**Published:** 2025-03-20

**Authors:** Youngae Jung, Seung Mi Lee, Jinhaeng Lee, Yeonjin Kim, Woojoo Lee, Ja Nam Koo, Ig Hwan Oh, Kue Hyun Kang, Byoung Jae Kim, Sun Min Kim, Jeesun Lee, Ji Hoi Kim, Yejin Bae, Sang Youn Kim, Gyoung Min Kim, Sae Kyung Joo, Dong Hyeon Lee, Joon Ho Moon, Bo Kyung Koo, Sue Shin, Errol R. Norwitz, Geum-Sook Hwang, Joong Shin Park, Won Kim

**Affiliations:** 1 Integrated Metabolomics Research Group, Metropolitan Seoul Center, Korea Basic Science Institute, University-Industry Cooperate Building, 150 Bugahyeon-ro, Seodaemun-gu, Seoul, 03759 Republic of Korea; 2 Department of Obstetrics and Gynecology, Seoul National University College of Medicine, 101 Daehak-ro, Jongno-gu, Seoul, 03080 Republic of Korea; 3 Innovative Medical Technology Research Institute, Seoul National University Hospital, Seoul, Republic of Korea; 4 Medical Big Data Research Center & Institute of Reproductive Medicine and Population, Medical Research Center, Seoul National University, Seoul, Republic of Korea; 5 Department of Public Health Sciences, Graduate School of Public Health, Seoul National University, Seoul, Republic of Korea; 6 Seoul Women’s Hospital, Incheon, Republic of Korea; 7 Department of Obstetrics and Gynecology, Seoul Metropolitan Government Seoul National University Boramae Medical Center, Seoul, Republic of Korea; 8 Department of Chemistry, Sungkyunkwan University, Suwon, Republic of Korea; 9 Department of Radiology, Seoul National University College of Medicine, Seoul, Republic of Korea; 10 Department of Radiology, Yonsei University College of Medicine, Seoul, Republic of Korea; 11 Department of Internal Medicine, Seoul National University College of Medicine, Seoul, Republic of Korea; 12 Division of Gastroenterology and Hepatology, Department of Internal Medicine, Seoul Metropolitan Government Seoul National University Boramae Medical Center, 20 Boramae-ro 5-gil, Dongjak-gu, Seoul, 07061 Republic of Korea; 13 Department of Internal Medicine, Bundang Seoul National University Hospital, Seongnam-si, Gyeonggi-do Republic of Korea; 14 Department of Laboratory Medicine, Seoul National University College of Medicine, Seoul, Republic of Korea; 15 Department of Laboratory Medicine, Seoul Metropolitan Government Seoul National University Boramae Medical Center, Seoul, Republic of Korea; 16 Department of Obstetrics and Gynecology, Tufts University School of Medicine, Boston, MA USA; 17 College of Pharmacy, Chung-Ang University, Seoul, Republic of Korea

**Keywords:** Metabolomics, Gestational diabetes mellitus, Hepatic steatosis, Mediation analysis, Early biomarker

## Abstract

**Background:**

This study aims to identify early metabolomic biomarkers of gestational diabetes mellitus (GDM) and evaluate their association with hepatic steatosis.

**Methods:**

We compared maternal serum metabolomic profiles between women who developed GDM (*n* = 118) and matched controls (*n* = 118) during the first (10–14 gestational weeks) and second (24–28 gestational weeks) trimesters using ultra-performance liquid chromatography coupled with mass spectrometry. Mediation analysis was performed to evaluate the mediating role of metabolic dysfunction-associated steatotic liver disease (MASLD) in the relationship between metabolites and subsequent development of GDM. A refined prediction model was developed to predict GDM using established clinical factors and selected metabolites.

**Results:**

Significant alterations in circulating metabolites, including amino acids, bile acids, and phospholipids, were observed in the GDM group compared to controls during early pregnancy. Mediation analysis revealed that several metabolites, including glycocholic acid (proportion mediated (PM) = 31.9%), butanoyl carnitine (PM = 25.7%), and uric acid (PM = 22.4%), had significant indirect effects on GDM incidence mediated by hepatic steatosis. The refined prediction model composed of clinical factors and selected metabolites in the first trimester demonstrated higher performance in predicting GDM development than the established prediction model composed solely of clinical factors (AUC, 0.85 vs. 0.63, *p* < 0.001).

**Conclusions:**

Women who developed GDM exhibited altered metabolomic profiles from early pregnancy, which showed a significant correlation with GDM, with MASLD as a mediator. Selected metabolomic biomarkers may serve as predictive markers and potential targets for early risk assessment and intervention in GDM.

**Graphical abstract:**

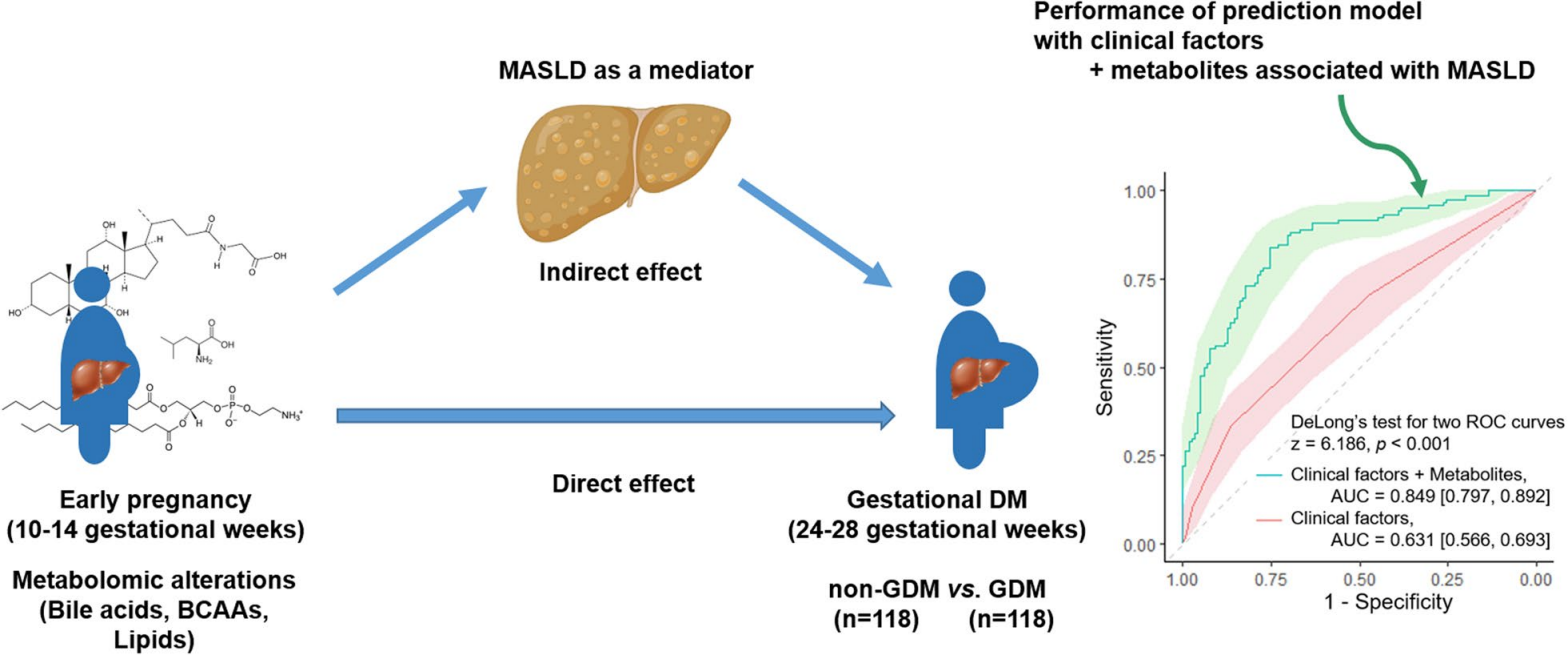

**Research insights:**

**What is currently known about this topic?:**

Gestational diabetes mellitus (GDM) is a common pregnancy complication with significant health risks. Early identification of women at high risk for GDM is crucial for timely intervention and improved outcomes.

**What is the key research question?:**

What alterations in circulating metabolites during early pregnancy are associated with subsequent GDM development?

Does metabolic dysfunction-associated steatotic liver disease (MASLD) mediate the association between specific metabolites and GDM risk?

**What is new?:**

Significant alterations in bile acids, amino acids, phosphatidylethanolamines, and phosphatidylinositols were observed in early pregnancy sera of women who later developed GDM. MASLD significantly mediated the effects of several metabolites on GDM risk, with mediation proportions ranging from 9.7 to 31.9%. A refined prediction model composed of clinical factors and metabolites significantly improved the performance in predicting GDM development.

**How might this study influence clinical practice?:**

These results provide new insights into early metabolic alterations associated with GDM development and highlight the potential mediating role of MASLD. This comprehensive metabolomic approach may contribute to the development of improved risk prediction models and targeted interventions for GDM prevention.

**Supplementary Information:**

The online version contains supplementary material available at 10.1186/s12933-025-02645-4.

## Background

Gestational diabetes mellitus (GDM) is a common pregnancy complication, affecting approximately 14% of pregnancies worldwide [1]. GDM is associated with adverse maternal and fetal outcomes during pregnancy and increased long-term health risks for both mothers and children [2–8]. Early identification of women at high risk for GDM is crucial for timely intervention and improved outcomes [9–11].

Current screening strategies for GDM, typically conducted between 24 and 28 weeks of gestation, may miss opportunities for early intervention [12, 13]. Recent studies have explored the potential of metabolomics to identify early biomarkers for GDM risk. Among metabolites in early pregnancy, selected metabolites have been used to develop prediction models for GDM [14, 15]. Additionally, emerging evidence suggests a link between metabolic dysfunction-associated steatotic liver disease (MASLD), formerly known as non-alcoholic fatty liver disease, and the development of GDM [12, 16–18].

Metabolomics offers a comprehensive approach to understanding the metabolic alterations that precede GDM diagnosis. Previous studies have demonstrated associations between various metabolites, including amino acids, lipids, and microbiota-related compounds, and subsequent GDM development [15, 19]. However, many of these studies have been limited by their focus on specific metabolite classes or use of single analytical techniques.

In this study, we aimed to conduct a comprehensive metabolomic analysis of serum samples from early pregnancy to identify potential biomarkers of GDM risk. We utilized a multi-platform approach, incorporating bile acid (BA) analysis, polar metabolite profiling, and lipidomics. Furthermore, we investigated the potential mediating role of MASLD in the relationship between circulating metabolites and GDM development. Finally, a refined prediction model was developed to predict GDM using clinical factors and selected predictive metabolites.

## Methods

### Study subjects

This study used data from the ongoing “Fatty Liver in Pregnancy” registry (NCT02276144), a prospective cohort study initiated in 2014 at Incheon Seoul Women’s Hospital and Seoul Metropolitan Government Seoul National University Boramae Medical Center in Korea. The study was approved by the Institutional Review Board of Seoul Metropolitan Government Seoul National University Boramae Medical Center (no. 26-2014-18) and the Public Institutional Review Board designated by the Ministry of Health and Welfare (no. P01-201404-BM-03). All participants provided informed consent.

**Fig. 1:**
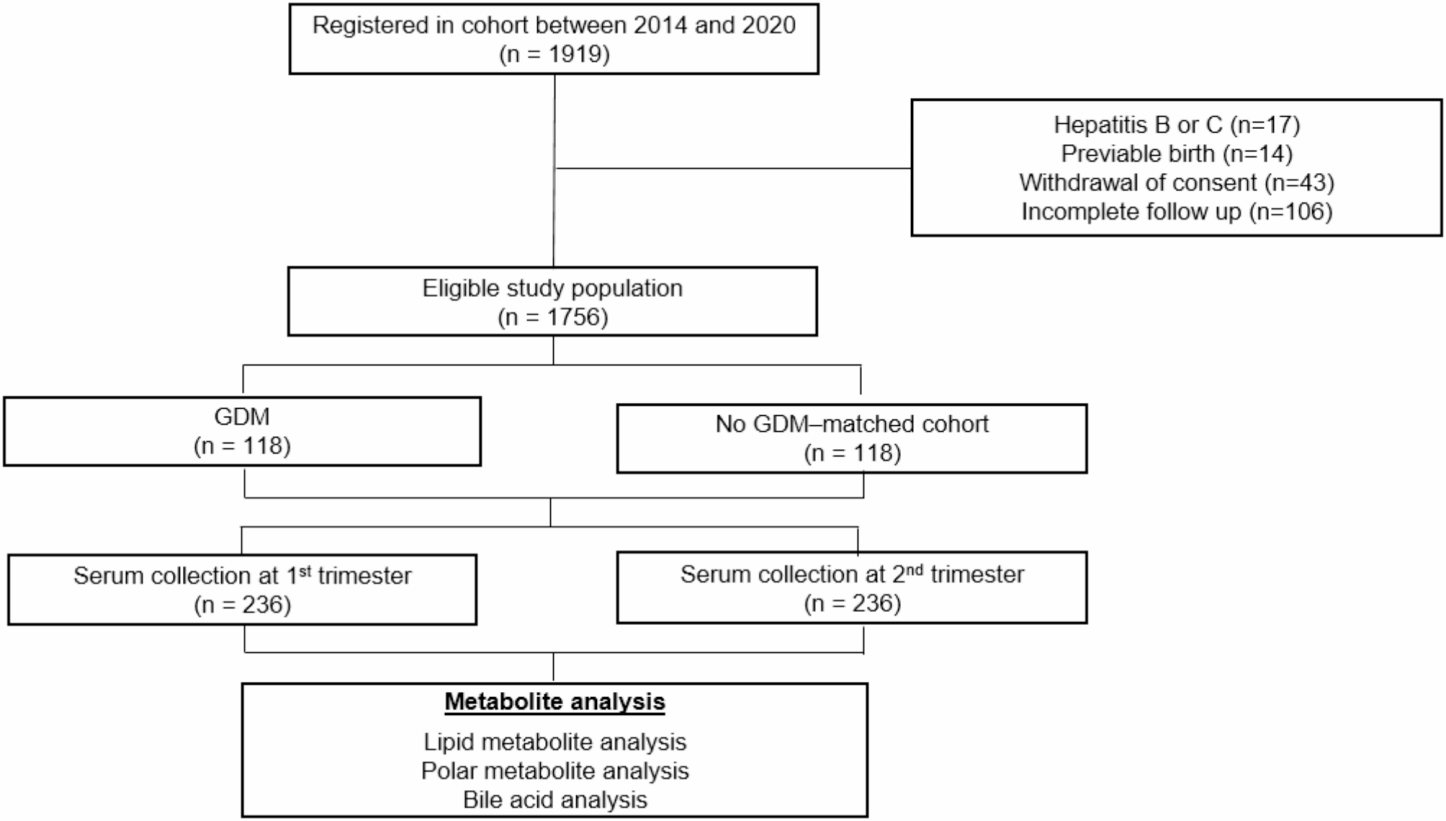
Schematic overviews of the study

Between 2014 and 2020, 1,936 women were enrolled in the cohort. Singleton pregnant women who visited the antenatal clinic in the first trimester were invited to participate (Fig. 1). Enrolled women were assessed for hepatic steatosis using liver ultrasound at 10–14 weeks of gestation and were followed until delivery. GDM was diagnosed using the two-step approach, following the recommendations of the American College of Obstetricians and Gynecologists [20]. For the 50 g oral glucose challenge test (GCT), plasma glucose was measured one hour after the glucose load, with ≥ 140 mg/dL considered positive. Women with a positive GCT underwent a 100 g oral GCT, and GDM was diagnosed if at least two values exceeded the thresholds (> 95 mg/dL fasting, > 180 mg/dL at 1 h, > 155 mg/dL at 2 h, and > 140 mg/dL at 3 h).

After applying exclusion criteria (viral hepatitis, previable birth, consent withdrawal, and incomplete follow-up), 1756 subjects remained eligible. From this population, we selected 118 women who developed GDM and 118 matched controls based on maternal age and parity.

### Clinical parameters and assessments

Participants were enrolled before 14 weeks of gestation. Baseline characteristics, including clinical and demographic information, were collected at enrollment via self-report questionnaire. At 10–14 weeks of gestation, participants underwent ultrasound examination to assess for the presence of hepatic steatosis, and fasting blood samples were collected. The presence of hepatic steatosis was evaluated based on the detection of bright echogenic patterns of the liver by ultrasonography. A semi-quantitative grading system (grade 0–3) was used to classify the severity of hepatic steatosis [21, 22].

The second blood sample was obtained at 24–28 weeks of gestation during the 50 g glucose challenge test. In this case-control study, we performed lipidomic analysis, polar metabolite analysis, and BA analysis on serum samples collected at both 10–14 weeks and 24–28 weeks of gestation. Results were analyzed in relation to subsequent GDM development.

### Serum BA analysis by UPLC/TQ MS

For serum BA analysis, 20 µL of serum samples were dispensed into a 96-well plate. All the samples were pooled in equal volumes to generate quality control (QC) samples, which were analyzed prior to sample acquisition and subsequently after every 11 samples. For protein precipitation, 80 µL of methanol was added to each well and vortexed for 10 min. After vortexing, the mixtures were incubated at 4 °C for 2 h, followed by centrifugation at 3300 × g for 20 min at 10 °C. 50 µL of supernatant were transferred into a new 96-well plate, and 50 µL of methanol: water (20:80, v/v) with internal standards was added to each well. BA Standard Mix 1– Unconjugated and BA Standard Mix 2– Conjugated (Cambridge Isotope Laboratories, Inc., USA) were used as internal standards. Serum extracts were analyzed by an ACQUITY UPLC system (Waters, Milford, MA, USA) coupled with a XEVO TQ-XS Triple Quadrupole MS system (Waters, Milford, MA, USA) in multiple reaction monitoring (MRM) mode. Chromatographic separation of BAs in the serum was performed using an ACQUITY UPLC BEH C18 column (2.1 × 50 mm, 1.7 μm; Waters) at 60 °C and a flow rate of 0.5 mL/min. The binary mobile phases comprised 5 mM ammonium acetate in water (solvent A) and methanol: acetonitrile (50:50 v/v, solvent B). The gradient was as follows: 35–40% B from 0 to 3.5 min, 40–45% B from 3.5 to 4.0 min, 45–50% B from 4.0 to 4.8 min, 50–55% B from 4.8 to 5.5 min, 55–80% B from 5.5 to 6.2 min, 80–85% B from 6.2 to 6.4 min, 85% B from 6.4 to 7.0 min, 85–35% B from 7.0 to 7.01 min, and 35% B from 7.01 to 9.0 min to equilibrate. Retention times and MRM transitions of each BA are shown in Supplementary Table S1. Measured BA concentrations below the limit of quantification (LOQ) were imputed as the LOQ of the respective BA. In the case of BA concentrations in which 30% or more are below the LOQ, those BAs, such as LCA, GLCA, TLCA and TUDCA, were excluded from further analysis.

### Serum semi-target analysis by UPLC/TQ MS

For serum polar metabolite analysis, 20 µL of serum samples were dispensed into a 96-well plate. All the samples were pooled in equal volumes to generate quality control (QC) samples, which were analyzed prior to sample acquisition and after every 11 samples. For protein precipitation, 80 µL of methanol was added to each well and vortexed for 10 min. After vortexing, the mixtures were incubated at 4 °C for 2 h, followed by centrifugation at 3300 × g for 20 min at 10 °C. Then, 50 µL of supernatant was transferred into a new 96-well plate and 50 µL of methanol: water (20:80 v/v) with internal standards was added to each well. A mixed solution of betaine D_11_, glutamate ^13^C, leucine ^13^C_6_, octanoyl carnitine ^13^C, phenylalanine ^13^C_6_, succinate ^13^C_2,_ taurine ^13^C, and uridine ^13^C was used as the internal standards. Serum semi-target analysis was conducted by 1290 Infinity LC (Agilent Technologies, USA) coupled to a 6490 Triple Quadrupole MS system (Agilent Technologies, USA) in the dynamic multiple reaction monitoring (dMRM) mode. Chromatographic separation of polar metabolites was performed using a Scherzo SM-C18 column (2.0 × 100 mm, 3 μm; Imtakt) at 25 °C and a flow rate of 0.2 mL/min. The binary mobile phases comprised 0.1% (v/v) formic acid in water (solvent A) and 0.1% (v/v) formic acid in methanol (solvent B). The gradient was as follows: 5% B from 0 to 3.0 min, 5–100% B from 3.0 to 14.0 min, 100% B from 14.0 to 16.0 min, 100–5% B from 16.0 to 17.0 min, and 5% B from 17.0 to 20.0 min to equilibrate.

Information on selected metabolites and MRM transitions was described previously [23]. The MassHunter Workstation (Ver. B.06.00; Agilent Technologies) was used for data acquisition and analysis. After normalization by internal standards, three metabolites (5-methyltetrahydrofolate, adenosine, and riboflavin) with coefficients of variation > 30 in quality control samples were excluded. Then, total 62 metabolites were used for further statistical analysis.

### Serum lipidomic profiling by UPLC/QTOF MS

For serum lipidomic analysis, 20 µL of serum samples were dispensed into a 96-well plate. All the samples were pooled in equal volumes to generate quality control (QC) samples, which were analyzed prior to sample acquisition and after every 11 samples. For protein precipitation, 30 µL of water and 200 µL of isopropanol with internal standards were added to each well and vortexed for 10 min. After vortexing, the mixtures were incubated at 4 °C for 2 h followed by centrifugation at 3300 × g for 20 min at 10 °C. Then, 150 µL of supernatant were transferred into a new 96-well plate, and 20 µL of samples were transferred into new two conical plates (one each for positive and negative mode). 180 µL of isopropanol: water (80:20 v/v) was added to all wells. SPLASH Lipidomix (Avanti Polar Lipids, USA) was used as the internal standard. Lipidomic profiling analysis was conducted using an ACQUITY UPLC system (Waters, Milford, MA, USA) coupled with a XEVO G2-XS QTOF mass spectrometer equipped with an electrospray ionization (ESI) source (Waters, Milford, MA, USA). Chromatographic separation of lipids in the serum was performed using an ACQUITY UPLC CSH C18 column (2.1 × 100 mm, 1.7 μm; Waters) at 55 °C and a flow rate of 0.4 mL/min. The binary mobile phases comprised 10 mM ammonium formate and 0.1% (v/v) formic acid in acetonitrile: water (60:40 v/v, solvent A) and 0.1% (v/v) formic acid in isopropanol: acetonitrile (90:10 v/v, solvent B) for positive mode and 10 mM ammonium acetate in water: acetonitrile (60:40 v/v, solvent A) and isopropanol: acetonitrile (90:10 v/v, solvent B) for negative mode. The gradient was as follows: 40–43% B from 0 to 2 min, 43–50% B from 2 to 2.1 min, 50–54% B from 2.1 to 12 min, 54–70% B from 12 to 12.1 min, 70–99% B from 12.1 to 18 min, 99–40% B from 18 to 18.1 min, and 40% B from 18.1 to 20 min to equilibrate. The mass spectrometer was operated in positive and negative ion mode, and the mass range was set at 80–1500 *m*/*z* for analysis of the lipid extracts.

MS data produced from the sum of the total intensity of all detected peaks in a UPLC/QTOF-MS was processed with Progenesis QC (Waters) and normalized to QC samples. Features with coefficients of variation below 30% in pooled QC samples were selected to determine the precision of detection. Lipid metabolites were identified using several authentic standard compounds and online databases, such as the Human Metabolome Database (HMDB) (www.hmdb.ca), METLIN (https://metlin.scripps.edu) and LIPID MAPS (www.lipidmaps.org).

### Statistical analysis

Statistical analyses were performed using SPSS Statistics version 28.0 (IBM Corporation, Armonk, NY, USA), MedCalc version 23.0.5 (MedCalc Software, Ostend, Belgium), and R version 3.6.0 (http://www.r-project.org). To assess the normality of continuous variables, the Kolmogorov-Smirnov test was conducted. Continuous variables following a normal distribution are expressed as means ± standard deviations and compared using independent *t*-tests, while those not following a normal distribution are presented as medians (interquartile ranges) and compared using Mann-Whitney U-tests. Categorical variables are reported as numbers (percentages) and compared using chi-square tests. For metabolite data, all values are expressed as medians (interquartile ranges) and compared using ranked analysis of covariance (ANCOVA). *P*-values were obtained from ranked analysis of covariance (ANCOVA), with adjustments for maternal age, pre-pregnancy BMI, nulliparity, and family history of diabetes. For multiple testing, *p* values were adjusted with the Benjamini–Hochberg method. Spearman’s correlation was used to assess relationships between variables. Multiple logistic regression was performed to evaluate the association between circulating metabolites and GDM, adjusting for maternal age, pre-pregnancy body mass index (BMI), nulliparity, and family history of diabetes. In addition, causal mediation analysis was performed to evaluate the direct and indirect effects of circulating metabolites on GDM, with MASLD as a mediator. The R-package “mediation” [24] was used, adjusting for confounders such as maternal age, pre-pregnancy BMI, nulliparity, and family history of diabetes in both outcome and mediator models. A *p*-value < 0.05 was considered statistically significant for both direct and indirect effects. Receiver operating characteristic (ROC) curves were used to evaluate the predictive performance of clinical factors and selected metabolites in the first trimester. To establish the prediction models for GDM development, the risk score using clinical factors was calculated based on baseline clinical factors in the first trimester described previously [25]. Baseline clinical factors included overweight status, family history of DM, age, history of pregnancy complications, history of polycystic ovary syndrome (PCOS), issues with insulin or blood glucose regulation, and a history of high blood pressure, high cholesterol, and/or heart disease. The areas under the ROC curves (AUCs) were compared using the DeLong test [26]. Flow chart of the statistical analysis was presented in Supplementary Fig. 1.

## Results

### Baseline characteristics of study participants

Of the 1,936 pregnant women enrolled in the cohort, 236 women (118 women who developed GDM and 118 matched controls) participated in this study (Fig. 1). Table 1 presents the baseline characteristics of the study population. As expected, due to matching, maternal age and parity were similar between GDM cases and controls. Women who developed GDM were more likely to have a family history of type 2 diabetes mellitus and had higher pre-pregnancy BMI. At enrollment, GDM cases also demonstrated higher BMI and blood pressure compared to controls. Ultrasound examinations at 10–14 weeks of gestation revealed a higher frequency of steatotic liver disease in women who subsequently developed GDM (35.2% vs. 10.7%, *p* < 0.001). Laboratory tests showed that GDM cases had higher median values of aminotransferases, cholesterol, low-density lipoprotein cholesterol, triglycerides, and gamma-glutamyl transferase (GGT).

**Table 1:** Baseline clinical, biochemical, and metabolic features of study participants in the first trimester

Characteristics	non-GDM	GDM	*p*-value
	(*n* = 118)	(*n* = 118)	
*Baseline characteristics before and during pregnancy*			
Age (years)	33.2 ± 4.8	33.4 ± 4.7	0.753
Nulliparity	70 (59.3)	69 (58.5)	0.895
Family history of type 2 diabetes mellitus	24 (20.3)	45 (38.1)	0.003
History of prior gestational diabetes mellitus	2 (1.7)	7 (5.9)	0.089
History of prior hypertensive disease during pregnancy	2 (1.7)	4 (3.4)	0.408
Overt diabetes mellitus before pregnancy	0 (0)	0 (0)	NA
Chronic hypertension before pregnancy	5 (4.2)	5 (4.2)	0.989
Body mass index before pregnancy (kg/m^2^)	20.8 (19.34–23.4)	24.2 (21.5–28.3)	< 0.001
Waist circumference before pregnancy (cm)	72.0 (70.0–75.0)	74.0 (69.0–79.0)	0.136
*Baseline characteristics in the first trimester*			
Gestational age at sampling during the first trimester	11.5 (11.2–12.2)	12.3 (12.0–13.0)	< 0.001
Body mass index (kg/m^2^)	21.3 (19.7–23.4)	25.0 (21.6–28.2)	< 0.001
Waist circumference (cm)	82.5 (78.0–88.0)	83.0 (75.0–93.6)	0.395
Systolic blood pressure (mmHg)	113.4 ± 9.8	119.3 ± 12.5	< 0.001
Diastolic blood pressure (mmHg)	71.5 ± 8.2	72.5 ± 9.2	0.373
Steatotic liver disease by ultrasound at 10–14 weeks	12 (10.2)	43 (36.4)	< 0.001
*Laboratory results in the first trimester*			
Aspartate aminotransferase (IU/L)	15.0 (13.0–19.0)	15.0 (13.0–18.5)	0.602
Alanine aminotransferase (IU/L)	8.0 (6.0–12.0)	12.0 (9.8–18.0)	< 0.001
Total cholesterol (mg/dL)	166.5 (142.3–188.0)	187.0 (168.0–205.0)	< 0.001
High-density lipoprotein cholesterol (mg/dL)	63.5 ± 17.5	66.0 ± 14.8	0.264
Low-density lipoprotein cholesterol (mg/dL)	76.0 ± 25.6	88.3 ± 24.4	< 0.001
Triglycerides (mg/dL)	103.0 (88.5–131.3)	139.5 (113.3–178.9)	< 0.001
gamma-Glutamyl transferase (IU/L)	10.0 (7.8–14.0)	14.0 (10.0–19.0)	< 0.001

Data are presented as numbers (%), means ± SDs or medians (interquantile ranges).

NA, non-applicable.

*p*-value from chi-square, Student’s *t*-test or Mann-Whitney U test as appropriate.

### Association between circulating metabolites in the first trimester and GDM

BA, polar semi-targeted, and lipidomic analyses were performed on a total of 236 sera collected from 236 study participants in the first trimester before GDM diagnosis using UPLC/TQ-MS and UPLC/QTOF-MS. The quantified 11 BAs and 62 polar metabolites are summarized in Supplementary Tables S2 and S3. Lipidomic profiles encompassed all lipid classes: free fatty acids (FFAs), ceramides (Cers), sphingomyelins (SMs), lysophosphatidylcholines (LPCs), lysophosphatidylethanolamines (LPEs), phosphatidylcholines (PCs), phosphatidylethanolamines (PEs), phosphatidylinositols (PIs), diacylglycerols (DAGs), and triacylglycerols (TAGs). All annotated lipid metabolites are summarized in Supplementary Table S4.

**Fig. 2:**
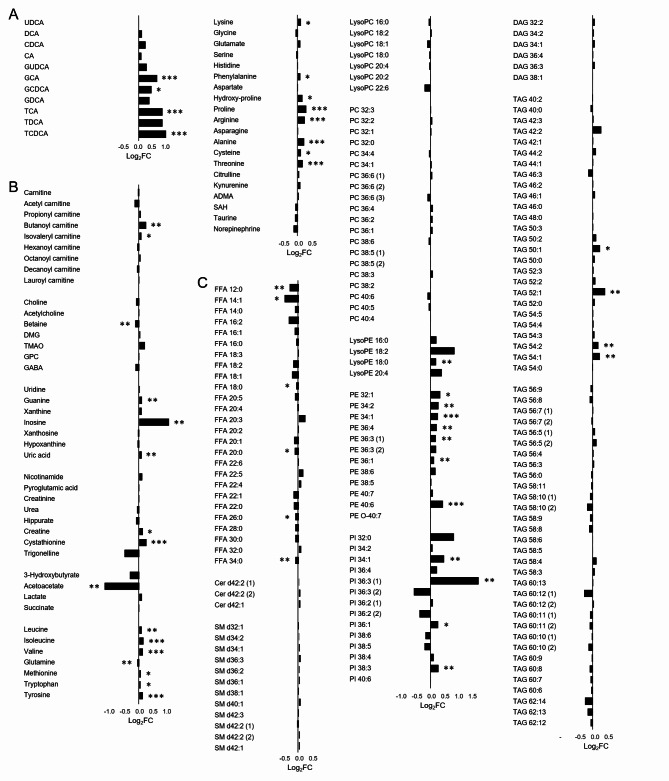
Alterations of circulating metabolites in serum samples from pregnant women during the first trimester. (**A**) bile acid (BA) analysis, (**B**) polar semi-targeted analysis, and (**C**) lipidomic analysis. Fold changes were calculated by dividing the median value of GDM group by the median value of non-GDM group. Adjusted *p*-values (*p*_*adj*_) were calculated using the Benjamini–Hochberg method from *p*-values obtained from ranked analysis of covariance (ANCOVA) test, with adjustments for maternal age, pre-pregnancy BMI, nulliparity, and family history of diabetes; ^*^p_*adj*_<0.05, ^**^p_*adj*_ <0.01, and ^***^p_*adj*_ <0.001

Comprehensive metabolomic analysis revealed significant alterations in diverse circulating metabolites in early pregnancy serum samples from women who later developed GDM (Fig. 2). After adjusting for potential confounders, the most distinctive changes were observed in BAs, amino acids, PEs, and PIs. BAs, particularly conjugated primary BAs such as glycocholate (GCA), glycochenodeoxycholate (GCDCA), taurocholate (TCA), and taurochenodeoxycholate (TCDCA), showed characteristic changes in the GDM group (Fig. 2A). Among polar metabolites, levels of several amino acids, including branched-chain amino acids (leucine, isoleucine, valine), aromatic amino acids (tyrosine), and others (proline, arginine, alanine, threonine), were elevated in the GDM group (Fig. 2B). Lipidomic analysis revealed significant alterations in FFAs, LysoPEs, PEs, PIs, and TAGs between the GDM and control groups (Fig. 2C). Notably, several PE species (PE 34:1, PE 36:1, PE 36:3, PE 36:4, PE 40:6) showed marked increases in the GDM group.

### Association between circulating metabolites in the first trimester and GDM risk factors

**Fig. 3:**
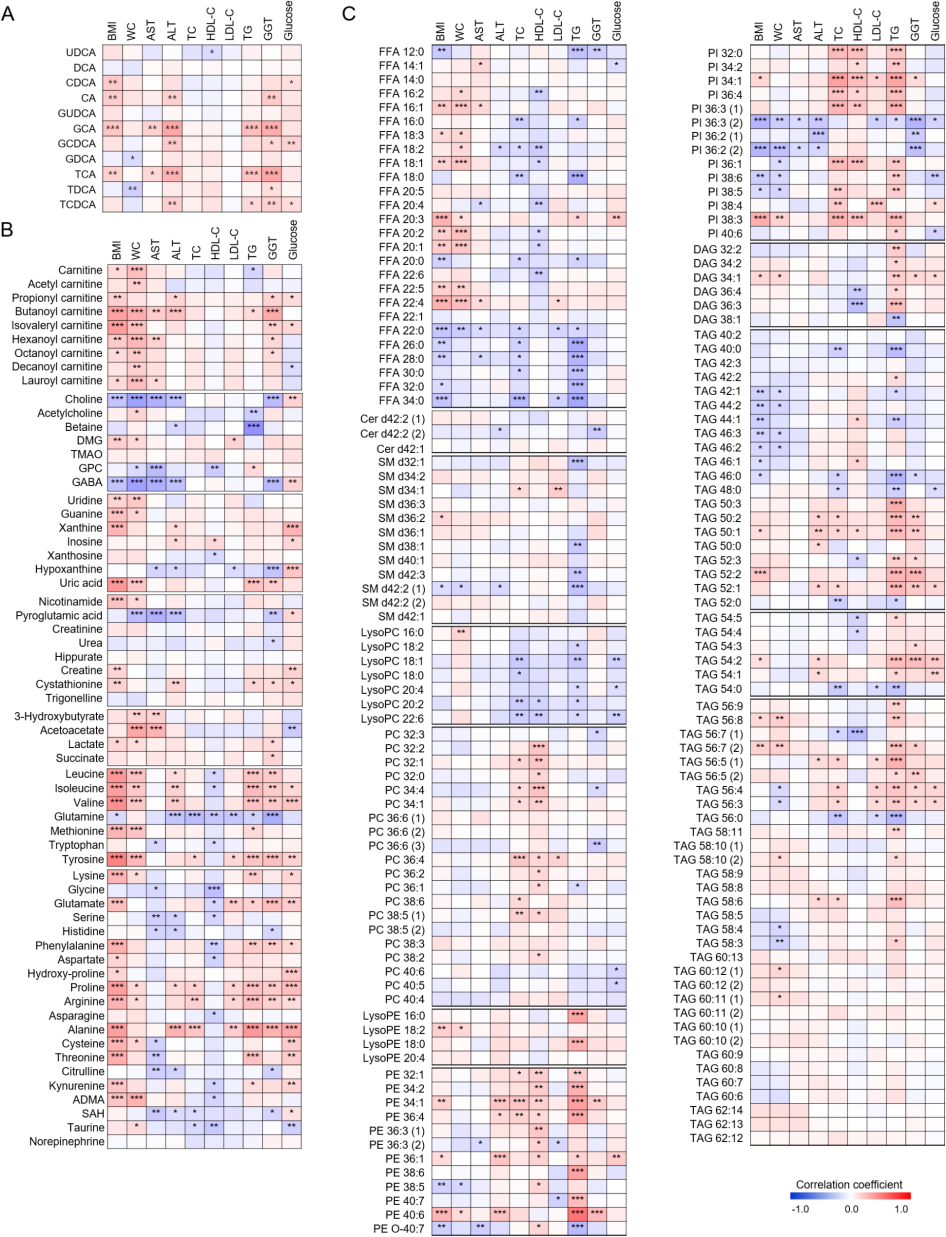
Correlations between circulating metabolite levels and laboratory results in serum samples from pregnant women during the first trimester. (**A**) bile acid (BA) analysis, (**B**) polar semi-targeted analysis, and (**C**) lipidomic analysis. Statistical significance in Spearman’s correlation heat maps is indicated with asterisks (^*^p < 0.05, ^**^p < 0.01, and ^***^p < 0.001)

Correlation analysis revealed significant associations between altered metabolites and established GDM risk factors (Fig. 3). Amino acids, particularly branched-chain amino acids (BCAAs) and tyrosine, showed strong correlations with waist circumference, triglycerides (TGs), and GGT. BA levels primarily correlated with TGs and fasting blood glucose, while PE and PI levels showed strong associations with TGs and total cholesterol. Consequently, metabolites exhibiting significant changes associated with GDM development commonly demonstrated strong correlations with TGs.

### Association between circulating metabolites in the first trimester and GDM based on MASLD status

**Fig. 4:**
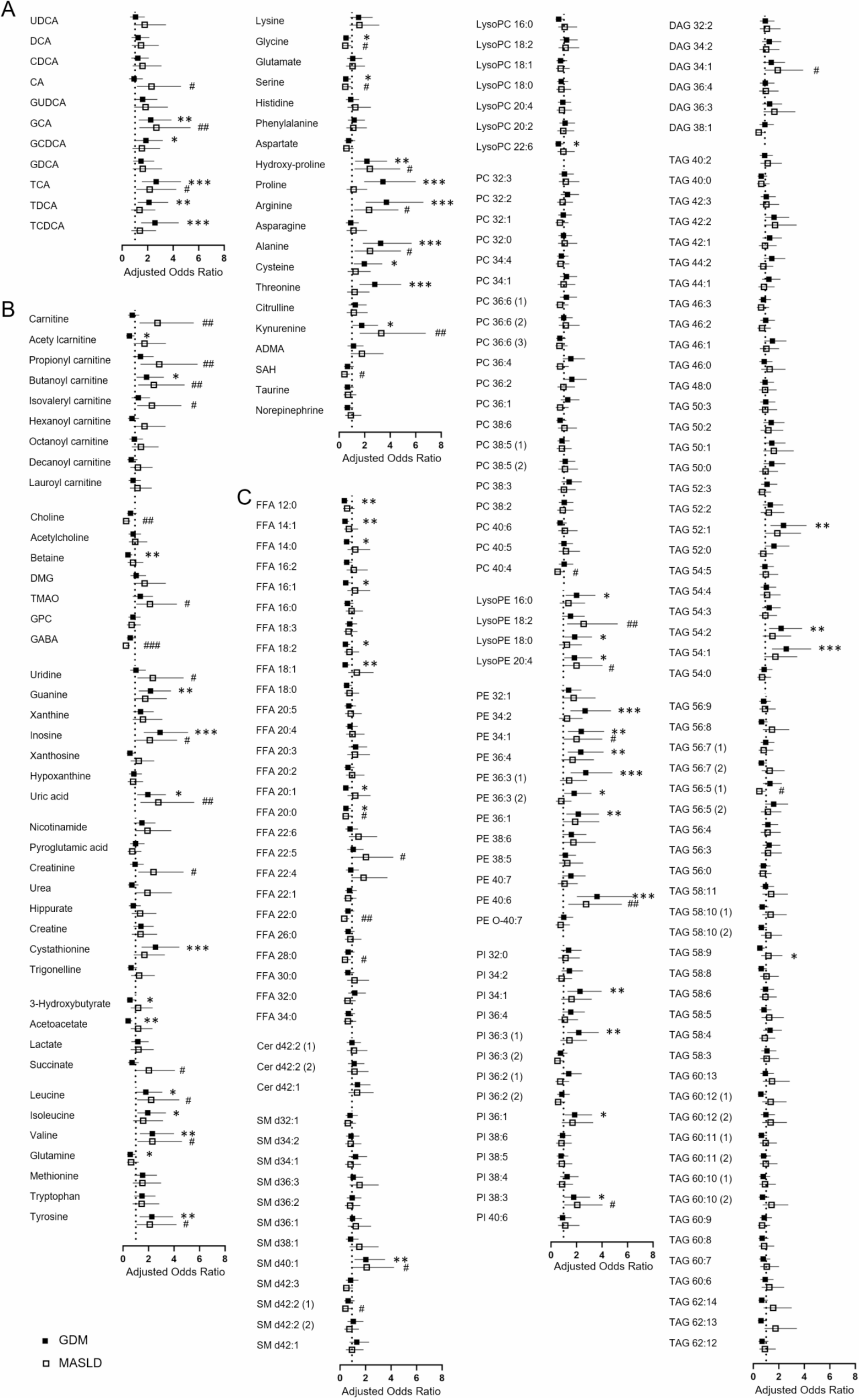
Associations between circulating metabolites and GDM / MASLD during the first trimester. (**A**) bile acid (BA) analysis, (**B**) polar semi-targeted analysis, and (**C**) lipidomic analysis. Multivariable–adjusted odds ratio, 95% CI and *p*-value were calculated from multiple logistic regression with adjustments for maternal age, pre-pregnancy BMI, nulliparity and family history of diabetes; ^*^p < 0.05, ^**^p < 0.01, and ^***^p < 0.001 for GDM and ^#^*p* < 0.05, ^##^*p* < 0.01, and ^###^*p* < 0.001 for MASLD

Multiple logistic regression analysis further elucidated the relationships between specific metabolites and the risk of GDM and MASLD (Fig. 4). Several conjugated BAs (GCA, GCDCA, TCA, TDCA, TCDCA) were associated with increased GDM risk, while CA, GCA, and TCA correlated with MASLD risk (Fig. 4A). Among polar metabolites, various amino acids (leucine, valine, tyrosine, hydroxyproline, arginine, alanine, kynurenine) were associated with increased risks of both GDM and MASLD (Fig. 4B). Interestingly, carnitine and some short-chain acylcarnitines showed a distinct relationship with MASLD risk. Additionally, certain lipid metabolites, particularly from FFA, PE, and PI classes, demonstrated stronger associations with GDM risk compared to MASLD risk (Fig. 4C). Mediation analysis revealed that MASLD significantly mediated the effects of several metabolites on GDM risk (Table 2). A total of 56 metabolites demonstrated a significant total effect on the relationship between metabolites and GDM (Supplementary Table S5). Conjugated primary BAs (GCA and TCA), acylcarnitine (butanoyl carnitine), purine metabolites (inosine and uric acid), and various amino acids showed significant indirect effects on GDM incidence mediated by MASLD, with mediation proportions ranging from 9.7 to 31.9%. Additionally, several lipids, such as PE 40:6, PI 38:3, and SM d40:1, showed significant mediation effects through MASLD. Pathway analysis from *MetaboAnalyst* (https://www.metaboanalyst.ca/) confirmed that these significant metabolites are linked to biological processes such as valine, leucine, and isoleucine biosynthesis, as well as primary BA metabolism (Supplementary Fig. S2).

**Table 2:** Mediation analysis on the association between circulating metabolites in the first trimester and GDM, with MASLD as a mediator

Metabolites	Total effect		Direct effect		Indirect effect		PM (%)
	β (95% CI)	*P*	β (95% CI)	*P*	β (95% CI)	*P*	
GCA	0.18 (0.06–0.30)	0.000	0.14 (0.03–0.26)	0.014	0.04 (0.00–0.09)	0.028	21.6
TCA	0.22 (0.10–0.34)	0.000	0.19 (0.07–0.30)	0.000	0.03 (− 0.00–0.08)	0.072	14.3
Butanoyl carnitine	0.16 (0.03–0.28)	0.014	0.11 (0.00–0.24)	0.066	0.04 (0.00–0.09)	0.028	25.9
Inosine	0.24 (0.12–0.35)	0.000	0.21 (0.10–0.33)	0.000	0.03 (0.00–0.07)	0.082	11.4
Uric acid	0.16 (0.04–0.27)	0.008	0.12 (0.00–0.23)	0.052	0.04 (0.00–0.09)	0.006	24.6
Leucine	0.14 (0.01–0.27)	0.026	0.11 (− 0.02–0.24)	0.102	0.03 (0.00–0.08)	0.078	22.0
Valine	0.20 (0.08–0.32)	0.000	0.16 (0.04–0.29)	0.004	0.04 (0.00–0.08)	0.056	17.1
Tyrosine	0.19 (0.07–0.32)	0.002	0.16 (0.04–0.29)	0.006	0.03 (0.00–0.08)	0.086	15.0
Glycine	−0.14 (− 0.26–0.01)	0.040	−0.10 (− 0.22–0.02)	0.100	−0.03 (− 0.08–0.00)	0.096	20.8
Hydroxy-proline	0.18 (0.06–0.30)	0.002	0.14 (0.02–0.26)	0.014	0.04 (0.00–0.08)	0.034	19.8
Arginine	0.29 (0.17–0.41)	0.000	0.26 (0.14–0.39)	0.000	0.03 (0.00–0.07)	0.074	9.7
Alanine	0.27 (0.15–0.39)	0.000	0.24 (0.12–0.36)	0.000	0.04 (0.00–0.08)	0.038	11.9
Kynurenine	0.13 (0.01–0.25)	0.032	0.09 (− 0.03–0.21)	0.152	0.04 (0.01–0.10)	0.006	31.9
PE 40:6	0.28 (0.17–0.40)	0.000	0.24 (0.12–0.36)	0.000	0.04 (0.01–0.09)	0.010	14.5
PI 38:3	0.14 (0.02–0.26)	0.024	0.10 (− 0.01–0.22)	0.094	0.03 (0.00–0.08)	0.098	22.7
SM d40:1	0.17 (0.05–0.29)	0.006	0.14 (0.03–0.26)	0.014	0.03 (0.00–0.07)	0.082	16.8

β, 95% CI, *p*-value, and PM were calculated from mediation analysis with adjustments for maternal age, pre-pregnancy BMI, nulliparity, and family history of diabetes

### Association between circulating metabolites in the second trimester and GDM

**Fig. 5:**
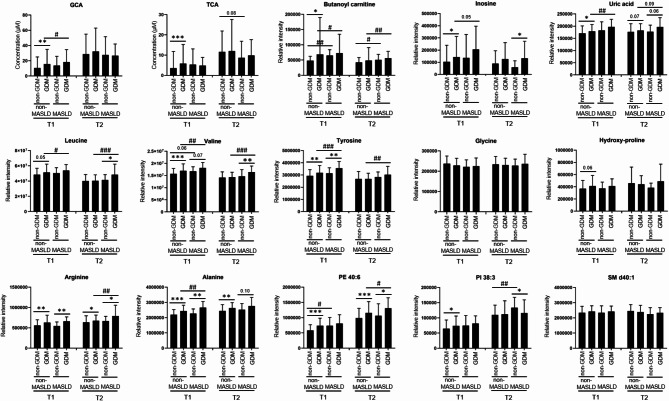
Alterations in circulating metabolites according to MASLD status in serum samples from pregnant women during the first and second trimesters. *P*-values were obtained from the ranked analysis of covariance (ANCOVA) test, with adjustments for maternal age, pre-pregnancy BMI, nulliparity and family history of diabetes; ^*^p < 0.05, ^**^p < 0.01, and ^***^p < 0.001 for GDM and ^#^*p* < 0.05, ^##^*p* < 0.01, and ^###^*p* < 0.001 for MASLD

To assess metabolic changes after GDM diagnosis, we also conducted BA, polar semi-targeted, and lipidomic analyses on sera collected from identical 236 study subjects in the second trimester. In the second trimester, women who developed GDM had higher values of BMI, AST, and TGs, with lower values of HDL-C than those who did not (Supplementary Table S6). The association between circulating metabolites and GDM risk differed based on MASLD status and gestational age (Fig. 5). Many metabolites showed further increases in GDM cases with concurrent MASLD at both the first and second trimesters.

### Prediction models for GDM development

**Fig. 6:**
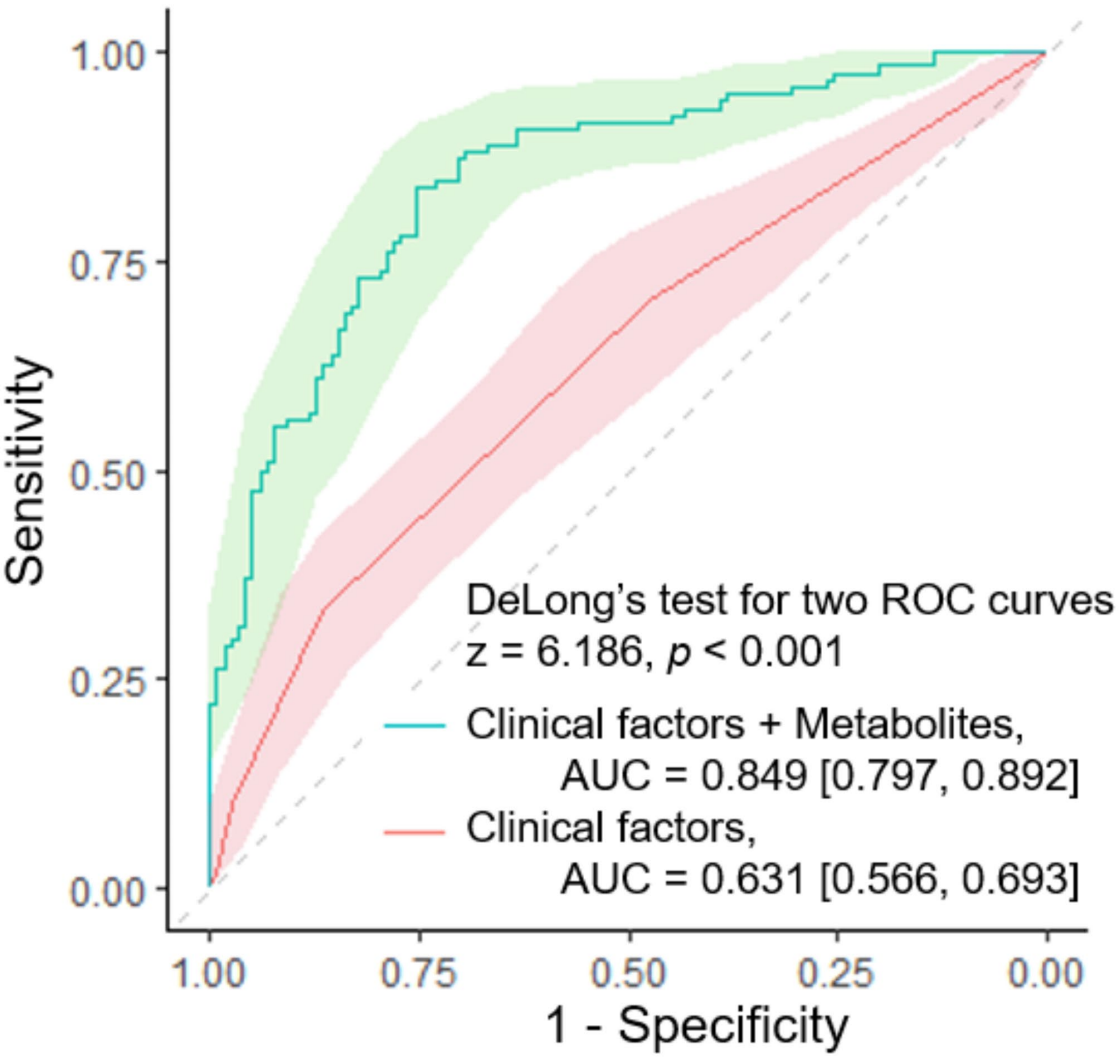
Prediction model evaluation using receiver operating characteristic (ROC) curves

A refined prediction model was developed incorporating both clinical factors and selected metabolites from the first trimester. Metabolites were selected based on their significant effects on GDM risk mediated by MASLD, including the following 16 metabolites: GCA, TCA, butanoyl carnitine, inosine, uric acid, leucine, valine, tyrosine, glycine, hydroxyl-proline, arginine, alanine, kynurenine, PE 40:6, PI 38:3, and SM d40:1. This model demonstrated significantly improved performance in predicting GDM development compared to the established prediction model based solely on clinical factors (AUC 0.85 vs. 0.63, *p* < 0.001, Fig. 6). The inclusion of metabolomic biomarkers significantly enhanced the model’s ability to identify women at high risk for GDM early in pregnancy.

## Discussion

### Main findings of the current study

This comprehensive metabolomic study identified prospective alterations in various metabolite classes during early pregnancy that were associated with subsequent GDM development. We observed that specific BAs, amino acids, and lipids were linked to both GDM and MASLD risk. Furthermore, mediation analysis suggested that certain circulating metabolites may influence GDM development through MASLD-related pathways.

### Metabolic profiling and the risk of GDM

Our findings aligned with previous studies demonstrating associations between amino acids, lipids, and purine metabolites in early pregnancy and GDM incidence [19, 27]. However, our multi-platform approach provided a more comprehensive metabolomic profile, revealing additional potential biomarkers and metabolic pathways involved in GDM pathogenesis.

The observed alterations in amino acid levels, particularly branched-chain amino acids (BCAAs), were consistent with their established role in insulin resistance and pancreatic β-cell dysfunction [28]. Elevated BCAA levels have been consistently associated with insulin resistance across various populations, including normoglycemic adults, obese individuals, and those with prediabetes or diabetes [28]. Our results suggested that increased BCAA levels in early pregnancy may indicate underlying insulin resistance and impaired β-cell function in women who later developed GDM.

Conversely, decreased levels of glutamine, glycine, and serine were also linked to an increased risk of GDM. While the literature has reported inconsistent findings regarding the relationship between amino acid levels and GDM, these discrepancies might be due to variations in genetic background, dietary intake, sample collection time, and environmental factors [19, 27].

Interestingly, we found that several amino acids, including BCAAs, exhibited significant mediation effects through MASLD. This finding aligns with previous research demonstrating altered amino acid profiles in MASLD patients, independent of obesity or insulin resistance [29]. The greater changes observed in GDM cases with concurrent MASLD suggested that pregnant women with MASLD may be at particularly high risk for GDM development.

Our study also identified novel associations between BA metabolism and GDM risk. While previous research on BAs in GDM has been limited and inconsistent [30, 31], our findings suggested that elevated levels of conjugated primary BAs, particularly GCA and TCA, in early pregnancy may contribute to disturbances in hepatic BA homeostasis and increase GDM risk. This is supported by recent evidence linking intrahepatic cholestasis of pregnancy, characterized by aberrant BA metabolism, to an increased incidence of GDM [32, 33]. Furthermore, our results indicated that chemically distinct BAs may exert varying effects on hyperglycemia across different species.

In the current study, elevated levels of uric acid, the end product of purine metabolism, were associated with increased risks of both GDM and MASLD, consistent with previous studies [34, 35]. Uric acid has been linked to insulin resistance, inflammation, and oxidative stress [36, 37], all of which may contribute to the pathogenesis of both GDM and MASLD.

Our findings regarding altered lipid profiles, particularly PEs and PIs, provided new insights into the lipid metabolism disturbances associated with GDM. Elevated levels of specific PE and PI species in early pregnancy may reflect underlying insulin resistance and altered lipid metabolism [38, 39]. These observations are consistent with previous studies reporting dysregulated lipid profiles in women who later developed GDM [40].

### Altered metabolic profiling for Predicting GDM Risk in the context of hepatic steatosis

The development of a prediction model incorporating both clinical factors and selected metabolites represented a significant advancement in early GDM risk assessment. Our refined model demonstrated superior performance compared to established models based solely on clinical factors [25], with an AUC of 0.85 versus 0.63 (*p* < 0.001). This finding aligns with recent studies highlighting the potential of integrating metabolomic biomarkers with traditional risk factors to improve GDM prediction [41, 42]. Although external validation is necessary to confirm the model’s generalizability, identifying a comparable external cohort is particularly challenging due to the unique characteristics of our study population. Nevertheless, our results demonstrate that incorporating metabolic biomarkers enhances predictive performance. Future studies with independent cohorts will be essential to further validate and strengthen our findings.

### MASLD, GDM, and metabolites

Previous studies have consistently reported an association between hepatic steatosis and GDM [12, 16–18]. However, the underlying mechanisms linking hepatic steatosis to GDM risk remain unclear. One possibility is that these two metabolic conditions share a common underlying dysfunction, such as insulin resistance [43, 44]. Additionally, proinflammatory cytokines released from intra-abdominal visceral tissue might be transferred to the liver, promoting hepatic fat accumulation and ultimately leading to hepatic steatosis [45, 46]. This process, along with the release of adipokines and increased lipolytic activity, may make visceral adipose tissue more metabolically detrimental, contributing to both hepatic steatosis and GDM [47, 48].

The significant mediation effects of MASLD on the relationship between several metabolites and GDM risk highlight the complex interplay between hepatic steatosis and glucose metabolism during pregnancy. This finding supports the emerging concept of MASLD as a potential driver of metabolic dysfunction in pregnancy [17, 18], rather than merely a consequence. We observed stronger associations between certain metabolites and GDM risk in women with concurrent MASLD, suggesting that this subgroup may benefit from more intensive monitoring and intervention strategies, such as lifestyle modifications.

### Strengths and limitations of the current study

Our study has several strengths, including its prospective design, comprehensive metabolomic profiling approach, and inclusion of MASLD assessment. The use of multiple analytical platforms allowed for a more holistic view of the metabolic alterations preceding GDM development. Additionally, the mediation analysis provided novel insights into potential mechanistic pathways linking metabolic disturbances to GDM risk.

However, some limitations should be acknowledged. First, the relatively small sample size, particularly for MASLD cases, may limit the generalizability of our findings. Second, while we adjusted for several potential confounders, residual confounding cannot be ruled out. Third, the use of non-fasting samples during the oral glucose loading test in the second trimester may have influenced some metabolite levels, although this limitation was mitigated by consistent sampling conditions across all participants. Lastly, we opted for a 1:1 matching strategy to ensure a well-balanced comparison between cases and controls. However, a higher control-to-case ratio would have provided additional statistical power.

Future research should focus on external validation of our findings in larger, more diverse cohorts and explore longitudinal changes in metabolomic profiles throughout pregnancy. Additionally, mechanistic studies are needed to elucidate the biological pathways underlying the observed associations between specific metabolites, MASLD, and GDM development.

## Conclusions

In conclusion, our comprehensive metabolomic analysis identified novel early pregnancy biomarkers associated with subsequent GDM development and highlighted the potential mediating role of MASLD. These findings enhance our understanding of the complex metabolic perturbations preceding GDM and may inform the development of improved risk prediction models and targeted interventions. The integration of metabolomic biomarkers with clinical risk factors shows promise for enhancing early GDM risk assessment, potentially enabling more timely and personalized preventive strategies.

## Electronic supplementary material

Below is the link to the electronic supplementary material.

Supplementary Material 1.

## Data Availability

No datasets were generated or analysed during the current study.

## Abbreviations

ADMA: Asymmetric dimethylarginine
ALT: Alanine aminotransferase
AST: Aspartate aminotransferase
BA: Bile acid
BMI: Body mass index
CA: Cholate
CDCA: Chenodeoxycholate
Cer: Ceramide
95% CI: 95% confidence intervals
DAG: Diacylglycerol
DCA: Deoxycholate
DMG: Dimethylglycine
FC: Fold change
FFA: Free fatty acid
GABA: γ-aminobutyrate
GCA: Glycocholate
GCDCA: Glycochenodeoxycholate
GDCA: Glycodeoxycholate
GDM: Gestational diabetes mellitus
GGT: Gamma-glutamyl transferase
GLCA: Glycolithocholate
GPC: Sn-glycerol-3-phosphocholine
GUDCA: Glycoursodeoxycholate
HDL-C: High-density lipoprotein-cholesterol
LCA: Litocholate
LDL-C: Low-density lipoprotein-cholesterol
LysoPC: Lysophosphatidylcholine
LysoPE: Lysophosphatidylethanolamine
MASLD: Metabolic dysfunction-associated steatotic liver disease
MRM: Multiple reaction monitoring
OR: Odd ratio
PC: Phosphatidylcholine
PE: Phosphatidylethanolamine
PI: Phosphatidylinositol
SAH: S-adenosylhomocysteine
SM: Sphingomyelin
TAG: Triacylglycerol
TCA: Taurocholate
TCDCA: Taurochenodeoxycholate
TDCA: Taurodeoxycholate
TLCA: Taurolithocholate
TGs: Triglycerides
TMAO: Trimethylamine-*N*-oxide
UDCA: Ursodeoxycholate
