# Supplementary material for: Metabolomic profiling reveals early biomarkers of gestational diabetes mellitus and associated hepatic steatosis
Source: Cardiovasc Diabetol. 2025 Mar 20;24:125. doi: 10.1186/s12933-025-02645-4 (PMC11927189; doi:10.1186/s12933-025-02645-4)

**Metabolomic Profiling Reveals Early Biomarkers of Gestational Diabetes Mellitus and Associated Hepatic Steatosis**

Youngae Jung^1*^, Seung Mi Lee^2,3,4*^, Jinhaeng Lee^1^, Yeonjin Kim^5^, Woojoo Lee^5^, Ja Nam Koo^6^, Ig Hwan Oh^6^, Kue Hyun Kang^6^, Byoung Jae Kim^2,7^, Sun Min Kim^2,7^, Jeesun Lee^2^, Ji Hoi Kim^2^, Yejin Bae^1,8^, Sang Youn Kim^9^, Gyoung Min Kim^10^, Sae Kyung Joo^11,12^, Dong Hyeon Lee^11,12^, Joon Ho Moon^11,13^, Bo Kyung Koo^11,12^, Sue Shin^14,15^, Errol R. Norwitz^16^, Geum-Sook Hwang^1,17¶^, Joong Shin Park^2¶^, Won Kim^11,12¶^

* Both authors contributed equally to this work and serve as co-first authors.

^¶^ Both authors contributed equally to this work and serve as co-corresponding authors.

*^1^ Integrated Metabolomics Research Group, Metropolitan Seoul Center, Korea Basic Science Institute, Seoul, Republic of Korea*

*^2^ Department of Obstetrics and Gynecology, Seoul National University College of Medicine, Seoul, Republic of Korea*

*^3^ Innovative Medical Technology Research Institute, Seoul National University Hospital, Seoul, Republic of Korea.*

*^4^ Medical Big Data Research Center & Institute of Reproductive Medicine and Population, Medical Research Center, Seoul National University, Seoul, Republic of Korea.*

*^5^ Department of Public Health Sciences, Graduate School of Public Health, Seoul National University, Seoul, Republic of Korea.*

*^6^ Seoul Women's Hospital, Incheon, Republic of Korea*

*^7^ Department of Obstetrics and Gynecology, Seoul Metropolitan Government Seoul National University Boramae Medical Center, Seoul, Republic of Korea*

*^8^ Department of Chemistry, Sungkyunkwan University, Suwon, Republic of Korea*

*^9^ Department of Radiology, Seoul National University College of Medicine, Seoul, Republic of Korea*

*^10^ Department of Radiology, Yonsei University College of Medicine, Seoul, Republic of Korea*

*^11^ Department of Internal Medicine, Seoul National University College of Medicine, Seoul, Republic of Korea*

*^12^ Department of Internal Medicine, Seoul Metropolitan Government Seoul National University Boramae Medical Center, Seoul, Republic of Korea*

*^13^ Department of Internal Medicine, Bundang Seoul National University Hospital, Gyeonggi-do, Republic of Korea*

*^14^ Department of Laboratory Medicine, Seoul National University College of Medicine, Seoul, Republic of Korea;*

*^15^ Department of Laboratory Medicine, Seoul Metropolitan Government Seoul National University Boramae Medical Center, Seoul, Republic of Korea*

*^16^ Department of Obstetrics and Gynecology, Tufts University School of Medicine, Boston, MA, U.S.A.*

*^17^ College of Pharmacy, Chung-Ang University, Seoul, Republic of Korea*

^¶^**Corresponding authors**

**Geum-Sook Hwang, PhD**

Integrated Metabolomics Research Group, Metropolitan Seoul Center, Korea Basic Science Institute, University-Industry Cooperate Building, 150 Bugahyeon-ro, Seodaemun-gu, Seoul 03759, Republic of Korea; [gshwang@kbsi.re.kr](mailto:gshwang@kbsi.re.kr)

**Joong Shin Park, MD, PhD**

Department of Obstetrics & Gynecology, Seoul National University College of Medicine, 101 Daehak-ro, Jongno-gu, Seoul 03080, Republic of Korea; [jsparkmd@snu.ac.kr](mailto:jsparkmd@snu.ac.kr)

**Won Kim, MD, PhD**

Division of Gastroenterology and Hepatology, Department of Internal Medicine, Seoul Metropolitan Government Seoul National University Boramae Medical Center, 20 Boramae-ro 5-gil, Dongjak-gu, Seoul 07061, Republic of Korea; drwon1@snu.ac.kr

**SUPPLEMENTARY TABLE LIST**

Supplementary Table S1. Retention time and MRM transition of bile acids.

Supplementary Table S2. List of bile acids quantified in sera obtained from pregnant women in the first and second trimester.

Supplementary Table S3. List of polar metabolites quantified in sera obtained from pregnant women in the first and second trimester.

Supplementary Table S4. List of lipid metabolites identified in sera obtained from pregnant women in the first and second trimester.

Supplementary Table S5. Mediation analysis for circulating metabolites and GDM via MASLD.

Supplementary Table S6. Baseline clinical, biochemical, and metabolic features of study populations in the second trimester.

**Supplementary Table S1. Retention time and MRM transition of bile acids.**

| Bile acids | Retention time  (min) | Precursor ion  (*m/z*) | Product ion  (*m/z*) |
| --- | --- | --- | --- |
| CA | 3.24 | 407.27 | 343.28 |
| CA-d4 | 3.24 | 411.27 | 347.28 |
| GCA | 2.50 | 464.30 | 74.00 |
| GCA-d4 | 2.50 | 468.30 | 74.00 |
| TCA | 2.68 | 514.28 | 80.00 |
| TCA-d4 | 2.68 | 518.28 | 80.00 |
| CDCA | 5.20 | 391.29 | 391.29 |
| CDCA-d4 | 5.20 | 395.29 | 395.29 |
| GCDCA | 4.36 | 448.30 | 74.00 |
| GCDCA-d4 | 4.36 | 452.30 | 74.00 |
| TCDCA | 4.55 | 498.32 | 80.00 |
| TCDCA-d4 | 4.55 | 502.32 | 80.00 |
| DCA | 5.41 | 391.28 | 391.28 |
| DCA-d4 | 5.41 | 395.28 | 395.28 |
| GDCA | 4.69 | 448.31 | 74.00 |
| GDCA-d4 | 4.69 | 452.31 | 74.00 |
| TDCA | 4.85 | 498.30 | 80.00 |
| TDCA-d4 | 4.85 | 502.30 | 80.00 |
| UDCA | 2.53 | 391.27 | 391.27 |
| UDCA-d4 | 2.53 | 395.27 | 395.27 |
| GUDCA | 1.74 | 448.29 | 74.00 |
| GUDCA-d4 | 1.74 | 452.29 | 74.00 |
| TUDCA | 1.91 | 498.28 | 80.01 |
| TUDCA-d4 | 1.91 | 502.28 | 80.01 |
| LCA | 6.30 | 375.25 | 375.25 |
| LCA-d4 | 6.30 | 379.25 | 379.25 |
| GLCA | 5.78 | 432.25 | 74.00 |
| GLCA-d4 | 5.78 | 436.25 | 74.00 |
| TLCA | 5.88 | 482.25 | 80.00 |
| TLCA-d4 | 5.88 | 486.25 | 80.00 |

Abbreviations: MRM, multiple reaction monitoring; CA, cholate; GCA, glycocholate; TCA, taurocholate; CDCA, chenodeoxycholate; GCDCA, glycochenodeoxycholate; TCDCA, taurochenodeoxycholate; DCA, deoxycholate; GDCA, glycodeoxycholate; TDCA, taurodeoxycholate; UDCA, ursodeoxycholate; GUDCA, glycoursodeoxycholate; TUDCA, tauroursodeoxycholate; LCA, lithocholate; GLCA, glycolithocholate; TLCA, taurolithocholate.

**Supplementary Table S2. List of bile acids quantified in sera obtained from pregnant women in the first and second trimester.**

| **Metabolites** | **First trimester** | | | | | | **Second trimester** | | | | | |
| --- | --- | --- | --- | --- | --- | --- | --- | --- | --- | --- | --- | --- |
|  | **non-GDM (n=118)** | | **GDM  (n=118)** | | ***P*** | ***P_adj_*** | **non-GDM  (n=118)** | | **GDM  (n=118)** | | ***P*** | ***P_adj_*** |
| **UDCA** | 3.0 | (1.3 – 7.2) | 2.9 | (1.1 – 8.3) | 0.790 | 0.538 | 2.8 | (1.3 – 6.1) | 3.3 | (1.7 – 8.3) | 0.342 | 0.206 |
| **DCA** | 26.1 | (15.2 – 44.5) | 28.1 | (12.3 – 42.9) | 0.905 | 0.572 | 43.2 | (25.1 – 61.0) | 34.2 | (16.0 – 51.4) | 0.050 | 0.051 |
| **CDCA** | 12.9 | (5.4 – 28.1) | 15.1 | (6.4 – 30.3) | 0.221 | 0.267 | 13.3 | (7.9 – 27.1) | 23.7 | (9.6 – 39.4) | 0.020 | 0.026 |
| **CA** | 4.5 | (2.1 – 8.6) | 4.8 | (2.6 – 9.3) | 0.407 | 0.376 | 6.0 | (3.6 – 11.9) | 7.0 | (3.9 – 16.0) | 0.105 | 0.085 |
| **GUDCA** | 2.9 | (1.3 – 5.7) | 3.5 | (1.9 – 7.2) | 0.078 | 0.138 | 4.7 | (2.3 – 8.1) | 4.0 | (2.5 – 7.6) | 0.833 | 0.383 |
| **GCA** | 6.7 | (3.9 – 12.3) | 10.5 | (6.5 – 18.7) | <0.001 | <0.001 | 22.8 | (12.7 – 35.5) | 24.8 | (14.3 – 40.0) | 0.325 | 0.198 |
| **GCDCA** | 27.3 | (11.9 – 42.3) | 37.2 | (19.2 – 58.2) | 0.003 | 0.011 | 56.9 | (38.5 – 84) | 55.4 | (42.1 – 85.1) | 0.756 | 0.360 |
| **GDCA** | 9.8 | (4.5 – 19.1) | 12.7 | (5.3 – 21.5) | 0.254 | 0.289 | 34.9 | (18.7 – 60.7) | 29.4 | (16.2 – 44.4) | 0.049 | 0.050 |
| **TCA** | 1.7 | (0.6 – 3.8) | 3.1 | (1.6 – 6.0) | <0.001 | <0.001 | 8.4 | (4.4 – 13.6) | 7.9 | (4.7 – 13.7) | 0.853 | 0.388 |
| **TDCA** | 1.7 | (0.8 – 4.9) | 3.1 | (1.3 – 6.2) | 0.024 | 0.059 | 13.0 | (6.7 – 19.9) | 9.7 | (4.9 – 16.8) | 0.036 | 0.041 |
| **TCDCA** | 5.3 | (3.0 – 10.4) | 10.4 | (5.3 – 18.5) | <0.001 | <0.001 | 23.7 | (16.5 – 41.2) | 22.1 | (14.7 – 36.2) | 0.318 | 0.195 |

The values are expressed as the median of μM concentration (interquartile range).

*P*-values were obtained using the Ranked ANCOVA test with adjustments for maternal age, pre-pregnancy body mass index, nulliparity, and family history of diabetes.

*P_adj_*-values were calculated using the Benjamini–Hochberg method to adjust multiple testing.

Abbreviations: GDM, gestational diabetes mellitus; UDCA, ursodeoxycholate; DCA, deoxycholate; CDCA, chenodeoxycholate; CA, cholate; GUDCA, glycoursodeoxycholate; GCA, glycocholate; GCDCA, glycochenodeoxycholate; GDCA, glycodeoxycholate; TCA, taurocholate; TDCA, taurodeoxycholate; TCDCA, taurochenodeoxycholate.

**Supplementary Table S3. List of polar metabolites quantified in sera obtained from pregnant women in the first and second trimester.**

| **Metabolite** | **Precursor ion (m/z)** | **Product ion (m/z)** | **RT** | **Ion Species** | **1st trimester** | | | | | | **2nd trimester** | | | | | | |
| --- | --- | --- | --- | --- | --- | --- | --- | --- | --- | --- | --- | --- | --- | --- | --- | --- | --- |
|  |  |  |  |  | **non-GDM (n=118)** | | **GDM  (n=118)** | | ***P*** | ***P_adj_*** | **non-GDM  (n=118)** | | **GDM  (n=118)** | | ***P*** | ***P_adj_*** |  |
| Carnitine | 162.00 | 60.00 | 1.09 | [M+H]^+^ | 830.71 | (762.65 - 934.91) | 800.9 | (734.93 - 916.35) | 0.327 | 0.331 | 667.06 | (599.56 - 740.37) | 671.73 | (583.67 - 756.24) | 0.976 | 0.421 |  |
| Acetyl carnitine | 204.00 | 85.00 | 1.24 | [M+H]^+^ | 945.23 | (739.95 - 1154.57) | 839.69 | (677.07 - 1087.46) | 0.024 | 0.059 | 490.14 | (405.66 - 584.42) | 552.80 | (457.24 - 673.04) | <0.001 | 0.002 |  |
| Propionyl carnitine | 218.10 | 85.10 | 1.67 | [M+H]^+^ | 198.34 | (168.74 - 227.6) | 207.42 | (171.1 - 241.56) | 0.294 | 0.313 | 176.32 | (150.97 - 201.28) | 159.90 | (137.48 - 191.26) | 0.028 | 0.034 |  |
| Butanoyl carnitine | 232.10 | 85.10 | 3.26 | [M+H]^+^ | 46.82 | (38.77 - 57.09) | 55.97 | (43.42 - 72.97) | 0.001 | 0.005 | 40.60 | (33.68 - 51.18) | 45.65 | (36.22 - 55.69) | 0.059 | 0.056 |  |
| Isovaleryl carnitine | 246.10 | 85.00 | 6.32 | [M+H]^+^ | 28.32 | (20.9 - 33.85) | 30.05 | (24.36 - 37.96) | 0.016 | 0.044 | 23.09 | (18.88 - 30.13) | 23.03 | (19.89 - 29.36) | 0.747 | 0.357 |  |
| Hexanoyl carnitine | 260.20 | 85.00 | 8.01 | [M+H]^+^ | 43.14 | (29.25 - 55.78) | 41.04 | (29.73 - 58.71) | 0.928 | 0.578 | 13.31 | (10.09 - 17.41) | 17.74 | (11.3 - 23.82) | <0.001 | <0.001 |  |
| Octanoyl carnitine | 288.20 | 85.20 | 10.26 | [M+H]^+^ | 206.48 | (142.65 - 284.37) | 212.84 | (141.57 - 311.41) | 0.863 | 0.560 | 48.85 | (32.51 - 68.28) | 72.86 | (49.64 - 108.09) | <0.001 | <0.001 |  |
| Decanoyl carnitine | 316.20 | 85.00 | 11.68 | [M+H]^+^ | 254.9 | (161.11 - 347.07) | 241.33 | (159.05 - 344.55) | 0.867 | 0.561 | 55.15 | (36.01 - 81.71) | 82.19 | (53.71 - 124.11) | <0.001 | <0.001 |  |
| Lauroyl carnitine | 344.20 | 85.20 | 12.67 | [M+H]^+^ | 41.34 | (30.28 - 52.9) | 41.34 | (27.01 - 54.5) | 0.428 | 0.387 | 12.20 | (8.3 - 16.8) | 15.25 | (11.8 - 21.75) | <0.001 | <0.001 |  |
| Choline | 104.10 | 60.20 | 1.05 | [M+H]^+^ | 3946.66 | (3033.12 - 5305.17) | 3633.06 | (2554.66 - 5214.56) | 0.139 | 0.199 | 3611.29 | (3060.22 - 4261.14) | 4188.87 | (3358.39 - 5041.07) | <0.001 | <0.001 |  |
| Acetylcholine | 146.20 | 87.00 | 1.10 | [M+H]^+^ | 67.6 | (59.22 - 76.19) | 66.18 | (58.51 - 78.39) | 0.615 | 0.476 | 64.23 | (56.25 - 73.98) | 61.49 | (53.73 - 75.93) | 0.639 | 0.322 |  |
| Betaine | 118.10 | 58.10 | 1.25 | [M+H]^+^ | 3985.34 | (3498.06 - 4698.09) | 3602.37 | (3010.46 - 4391.4) | 0.001 | 0.005 | 3269.75 | (2819.31 - 3765.59) | 2843.93 | (2468.27 - 3410.39) | <0.001 | <0.001 |  |
| DMG | 104.10 | 58.10 | 1.23 | [M+H]^+^ | 341.21 | (290.61 - 392.29) | 351.5 | (302.96 - 411.86) | 0.286 | 0.308 | 276.68 | (236.78 - 319.5) | 295.83 | (249.6 - 351.23) | 0.040 | 0.044 |  |
| TMAO | 76.10 | 58.00 | 1.09 | [M+H]^+^ | 55.81 | (35.46 - 98.98) | 64.58 | (39.34 - 121.42) | 0.223 | 0.268 | 97.57 | (50.21 - 244.72) | 75.00 | (50.03 - 132.43) | 0.029 | 0.035 |  |
| GPC | 258.00 | 104.00 | 1.26 | [M+H]^+^ | 1874.07 | (1456.52 - 2456.1) | 1854.16 | (1391.53 - 2496.43) | 0.849 | 0.556 | 1730.32 | (1257.06 - 2258.39) | 1752.59 | (1425.83 - 2358.44) | 0.210 | 0.144 |  |
| GABA | 104.00 | 45.20 | 1.05 | [M+H]^+^ | 2422.99 | (1895.21 - 3314.81) | 2204.54 | (1560.03 - 3186.79) | 0.105 | 0.167 | 2197.83 | (1869.39 - 2672.93) | 2568.11 | (2112.86 - 3108.45) | <0.001 | <0.001 |  |
| Uridine | 243.10 | 110.10 | 2.66 | [M-H]^-^ | 2.09 | (1.83 - 2.36) | 2.12 | (1.77 - 2.4) | 0.919 | 0.576 | 1.84 | (1.56 - 2.13) | 1.87 | (1.67 - 2.17) | 0.380 | 0.222 |  |
| Guanine | 152.00 | 135.00 | 1.65 | [M+H]^+^ | 2.69 | (2.46 - 3.03) | 2.89 | (2.59 - 3.2) | 0.002 | 0.008 | 2.41 | (2.18 - 2.78) | 2.61 | (2.33 - 2.89) | 0.018 | 0.024 |  |
| Xanthine | 153.00 | 110.00 | 3.05 | [M+H]^+^ | 201.55 | (174.82 - 236.79) | 214.09 | (185.7 - 252.45) | 0.028 | 0.066 | 181.65 | (158.07 - 213.71) | 182.86 | (159.63 - 216.71) | 0.753 | 0.359 |  |
| Inosine | 269.10 | 137.10 | 4.50 | [M+H]^+^ | 50 | (16.51 - 144.92) | 106.68 | (30.49 - 217.03) | <0.001 | 0.005 | 50.48 | (19.55 - 103.4) | 75.70 | (20.92 - 186.64) | 0.051 | 0.051 |  |
| Xanthosine | 283.00 | 151.00 | 6.11 | [M-H]^-^ | 0.17 | (0.15 - 0.2) | 0.16 | (0.15 - 0.2) | 0.513 | 0.431 | 0.16 | (0.14 - 0.2) | 0.16 | (0.14 - 0.19) | 0.689 | 0.339 |  |
| Hypoxanthine | 137.20 | 110.00 | 2.51 | [M+H]^+^ | 625.44 | (506.5 - 828.84) | 599.16 | (487.04 - 752.76) | 0.402 | 0.373 | 609.37 | (468.04 - 772.48) | 551.09 | (442.15 - 665.97) | 0.036 | 0.041 |  |
| Uric acid | 167.00 | 124.00 | 2.50 | [M-H]^-^ | 171 | (150.94 - 191.14) | 181.82 | (168.33 - 206.91) | <0.001 | 0.002 | 174.53 | (159.04 - 191.74) | 184.66 | (164.49 - 204.94) | 0.007 | 0.013 |  |
| Nicotinamide | 123.00 | 80.10 | 2.00 | [M+H]^+^ | 32.37 | (23.89 - 43.34) | 35 | (28.03 - 44.16) | 0.156 | 0.214 | 27.95 | (19.58 - 39.71) | 25.96 | (18.86 - 33.62) | 0.141 | 0.105 |  |
| Pyroglutamic acid | 130.00 | 56.20 | 3.65 | [M+H]^+^ | 94.48 | (79.9 - 122.27) | 94.45 | (81.91 - 112.54) | 0.814 | 0.546 | 91.76 | (78.47 - 109.43) | 93.34 | (78.76 - 109.29) | 0.942 | 0.412 |  |
| Creatinine | 114.00 | 44.20 | 1.08 | [M+H]^+^ | 759.3 | (673.85 - 844.58) | 757.03 | (671.39 - 851.73) | 0.884 | 0.566 | 727.57 | (632.72 - 811.11) | 770.41 | (673.87 - 863.74) | 0.027 | 0.034 |  |
| Urea | 61.20 | 44.20 | 1.33 | [M+H]^+^ | 7299.06 | (6195.92 - 7874.41) | 6917.1 | (5917.98 - 8295.23) | 0.460 | 0.404 | 7199.37 | (6257.63 - 8083.48) | 6830.12 | (5829.72 - 7994.11) | 0.113 | 0.090 |  |
| Hippurate | 178.00 | 134.00 | 10.23 | [M-H]^-^ | 1.27 | (0.64 - 2.41) | 1.18 | (0.56 - 2.23) | 0.645 | 0.488 | 5.47 | (4.09 - 6.77) | 4.79 | (3.64 - 5.97) | 0.014 | 0.021 |  |
| Creatine | 132.10 | 44.20 | 1.21 | [M+H]^+^ | 3249.86 | (2849.3 - 3814.42) | 3580.46 | (2954.76 - 4203.85) | 0.015 | 0.042 | 4552.85 | (3746.58 - 5477.66) | 4677.02 | (3973.85 - 5520.56) | 0.305 | 0.190 |  |
| Cystathionine | 223.10 | 87.90 | 1.14 | [M+H]^+^ | 2.15 | (1.82 - 2.71) | 2.58 | (2.15 - 3.43) | <0.001 | <0.001 | 4.63 | (3.49 - 6.39) | 4.49 | (3.58 - 6.24) | 0.918 | 0.406 |  |
| Trigonelline | 138.10 | 92.10 | 1.28 | [M+H]^+^ | 3.61 | (1.83 - 5.71) | 2.49 | (1.82 - 5.27) | 0.216 | 0.263 | 11.44 | (7.81 - 22) | 8.30 | (5.06 - 12.89) | <0.001 | <0.001 |  |
| 3-Hydroxybutyrate | 103.00 | 59.00 | 2.71 | [M-H]^-^ | 15.17 | (8.14 - 28.49) | 11.96 | (7.62 - 24.54) | 0.206 | 0.256 | 5.47 | (3.96 - 7.3) | 8.27 | (5.94 - 13) | <0.001 | <0.001 |  |
| Acetoacetate | 101.00 | 57.00 | 3.38 | [M-H]^-^ | 0.14 | (0.05 - 0.35) | 0.06 | (0.01 - 0.23) | 0.002 | 0.008 | 0.11 | (0.04 - 0.19) | 0.06 | (0.01 - 0.17) | 0.010 | 0.016 |  |
| Lactate | 89.00 | 43.00 | 2.29 | [M-H]^-^ | 135.47 | (112.22 - 156.81) | 145.11 | (116.04 - 165.63) | 0.070 | 0.129 | 154.91 | (136.64 - 176.72) | 179.54 | (150.25 - 200.87) | <0.001 | <0.001 |  |
| Succinate | 117.00 | 73.00 | 2.99 | [M-H]^-^ | 3.27 | (2.91 - 3.8) | 3.26 | (2.88 - 3.76) | 0.945 | 0.582 | 2.87 | (2.47 - 3.33) | 2.90 | (2.5 - 3.31) | 0.841 | 0.385 |  |
| Leucine | 132.00 | 86.00 | 2.09 | [M+H]^+^ | 47610.69 | (42833.9 - 52216.3) | 50934.24 | (45859.39 - 55613.15) | <0.001 | 0.003 | 38844.60 | (35725.4 - 43498.57) | 41486.52 | (36589.06 - 47191.45) | 0.027 | 0.034 |  |
| Isoleucine | 132.00 | 69.00 | 1.96 | [M+H]^+^ | 16480.26 | (15070.08 - 18938.85) | 18341.12 | (16428.41 - 20493.81) | <0.001 | <0.001 | 13615.31 | (11982.17 - 15915.06) | 14878.98 | (13280.51 - 17181.42) | 0.003 | 0.006 |  |
| Valine | 118.00 | 72.00 | 1.41 | [M+H]^+^ | 15591.74 | (14323.32 - 17027.83) | 17125.51 | (15500.05 - 18704.88) | <0.001 | <0.001 | 13815.76 | (12540.1 - 15605.04) | 14908.06 | (13405.72 - 16216.37) | 0.013 | 0.019 |  |
| Glutamine | 147.00 | 84.00 | 1.19 | [M+H]^+^ | 9808.85 | (9187.81 - 10660.28) | 9333.76 | (8463.57 - 10289.51) | 0.001 | 0.006 | 8783.75 | (7881.79 - 9512.59) | 7887.96 | (7112.16 - 8403.59) | <0.001 | <0.001 |  |
| Methionine | 149.90 | 133.00 | 1.65 | [M+H]^+^ | 921.09 | (836.45 - 1014.32) | 961.47 | (890.97 - 1095.1) | 0.006 | 0.021 | 817.25 | (721.74 - 923.27) | 839.66 | (743.55 - 932.58) | 0.260 | 0.169 |  |
| Tryptophan | 205.00 | 188.00 | 7.02 | [M+H]^+^ | 9348.51 | (8338.47 - 10170.26) | 9701.39 | (8884.74 - 10545.86) | 0.013 | 0.038 | 8153.96 | (7459.81 - 9079.33) | 8334.76 | (7821.05 - 8822.46) | 0.878 | 0.395 |  |
| Tyrosine | 182.00 | 165.00 | 2.43 | [M+H]^+^ | 2907.86 | (2576.05 - 3279.42) | 3205.94 | (2992.44 - 3591.83) | <0.001 | <0.001 | 2682.94 | (2256.31 - 3007.37) | 2728.44 | (2463.67 - 3032.35) | 0.201 | 0.139 |  |
| Lysine | 147.10 | 84.20 | 0.93 | [M+H]^+^ | 258.92 | (231.09 - 297.64) | 275.28 | (247.16 - 316.45) | 0.009 | 0.027 | 260.92 | (227.46 - 297.41) | 268.45 | (238.64 - 304.61) | 0.491 | 0.268 |  |
| Glycine | 75.90 | 30.10 | 1.14 | [M+H]^+^ | 231.64 | (206.86 - 259.79) | 219.98 | (204.96 - 244.22) | 0.079 | 0.140 | 231.95 | (211.65 - 252.16) | 230.45 | (216.15 - 253.28) | 0.724 | 0.350 |  |
| Glutamate | 148.00 | 84.00 | 1.28 | [M+H]^+^ | 3003.68 | (2517.53 - 3527.84) | 3152.18 | (2701.04 - 3683.31) | 0.031 | 0.072 | 3024.83 | (2632.68 - 3463.4) | 3341.30 | (2918.79 - 3835.8) | <0.001 | 0.001 |  |
| Serine | 106.00 | 60.00 | 1.18 | [M+H]^+^ | 1453.54 | (1282.77 - 1595.51) | 1397.2 | (1279.17 - 1552.1) | 0.185 | 0.240 | 1443.03 | (1329.49 - 1607.66) | 1457.37 | (1357.1 - 1626.12) | 0.311 | 0.192 |  |
| Histidine | 156.10 | 110.20 | 0.94 | [M+H]^+^ | 79.38 | (72.84 - 86.19) | 78.88 | (71.74 - 84.86) | 0.477 | 0.413 | 80.58 | (72.92 - 88.2) | 81.82 | (74.65 - 89.92) | 0.319 | 0.196 |  |
| Phenylalanine | 166.00 | 120.00 | 4.10 | [M+H]^+^ | 23202.45 | (19883.2 - 26481.65) | 24438.46 | (21634.74 - 27830.9) | 0.007 | 0.024 | 23631.22 | (20785.02 - 26264.32) | 25044.56 | (22614.31 - 29085.22) | 0.003 | 0.007 |  |
| Aspartate | 134.10 | 74.00 | 1.40 | [M+H]^+^ | 688.5 | (581.77 - 841.75) | 682.9 | (616.19 - 809.7) | 0.855 | 0.558 | 733.79 | (636.61 - 861.32) | 773.57 | (674.26 - 886.31) | 0.064 | 0.059 |  |
| Hydroxy-proline | 132.00 | 86.10 | 1.23 | [M+H]^+^ | 340.51 | (276.4 - 420.99) | 375.23 | (319.22 - 468.17) | 0.007 | 0.023 | 399.28 | (321.2 - 470.15) | 426.67 | (349.62 - 500.82) | 0.073 | 0.065 |  |
| Proline | 116.00 | 70.00 | 1.29 | [M+H]^+^ | 8763.25 | (7983.59 - 10246.94) | 10529.27 | (9304.23 - 11825.99) | <0.001 | <0.001 | 9642.87 | (8195.14 - 11379.65) | 10087.27 | (8866.72 - 11313.77) | 0.195 | 0.136 |  |
| Arginine | 175.10 | 70.30 | 1.07 | [M+H]^+^ | 536.78 | (474.9 - 636.76) | 622.33 | (545.82 - 718.66) | <0.001 | <0.001 | 618.15 | (546.52 - 726) | 683.41 | (611.52 - 758.04) | <0.001 | <0.001 |  |
| Asparagine | 133.00 | 74.00 | 1.17 | [M+H]^+^ | 352.37 | (326.77 - 403.81) | 352.72 | (327.55 - 397.28) | 0.872 | 0.563 | 409.23 | (354.25 - 454.71) | 382.11 | (348.89 - 425.86) | 0.016 | 0.022 |  |
| Alanine | 90.00 | 44.20 | 1.16 | [M+H]^+^ | 2191.15 | (1955.49 - 2437.68) | 2519.3 | (2256.36 - 2718.96) | <0.001 | <0.001 | 2476.68 | (2190.53 - 2715.5) | 2640.95 | (2444.44 - 2914.42) | <0.001 | <0.001 |  |
| Cysteine | 122.00 | 59.00 | 1.19 | [M+H]^+^ | 3.92 | (3.65 - 4.37) | 4.18 | (3.84 - 4.6) | 0.003 | 0.012 | 4.71 | (4.21 - 5.31) | 4.91 | (4.34 - 5.48) | 0.054 | 0.053 |  |
| Threonine | 120.10 | 73.90 | 1.19 | [M+H]^+^ | 1399.96 | (1246.4 - 1585.47) | 1550.95 | (1358.52 - 1769.86) | <0.001 | <0.001 | 1838.14 | (1661.09 - 2103.76) | 1898.66 | (1674.47 - 2152.88) | 0.338 | 0.204 |  |
| Citrulline | 176.00 | 159.00 | 1.22 | [M+H]^+^ | 505.72 | (457.6 - 601.05) | 517.7 | (463.03 - 582.05) | 0.938 | 0.580 | 422.23 | (361.07 - 507.93) | 374.17 | (332.25 - 460.23) | 0.004 | 0.008 |  |
| Kynurenine | 209.00 | 94.00 | 3.93 | [M+H]^+^ | 52.32 | (45.51 - 61.06) | 55.24 | (48.37 - 62.41) | 0.035 | 0.079 | 53.39 | (46.57 - 61.14) | 54.51 | (47.83 - 61.55) | 0.610 | 0.312 |  |
| ADMA | 203.10 | 116.00 | 1.11 | [M+H]^+^ | 24.32 | (21.39 - 26.73) | 24.82 | (22.27 - 27.2) | 0.262 | 0.294 | 27.89 | (25.74 - 31.46) | 28.27 | (25.85 - 32.16) | 0.581 | 0.302 |  |
| SAH | 385.00 | 136.10 | 2.28 | [M+H]^+^ | 1.42 | (1.11 - 1.67) | 1.33 | (1.07 - 1.55) | 0.203 | 0.254 | 1.40 | (1.18 - 1.63) | 1.39 | (1.16 - 1.61) | 0.603 | 0.310 |  |
| Taurine | 126.00 | 108.00 | 1.25 | [M+H]^+^ | 148.03 | (121.17 - 185.94) | 138.16 | (103.76 - 172.01) | 0.038 | 0.083 | 141.79 | (114.03 - 175.96) | 129.49 | (84.56 - 159.69) | 0.002 | 0.004 |  |
| Norepinephrine | 152.10 | 107.00 | 1.15 | [M+H]^+^ | 0.46 | (0.35 - 0.62) | 0.41 | (0.34 - 0.66) | 0.614 | 0.475 | 0.47 | (0.33 - 0.57) | 0.47 | (0.35 - 0.61) | 0.420 | 0.238 |  |

The values are expressed as the median/1,000 (interquartile range).

*P*-values were obtained using the Ranked ANCOVA test with adjustments for maternal age, pre-pregnancy body mass index, nulliparity, and family history of diabetes.

*P_adj_*-values were calculated using the Benjamini–Hochberg method to adjust multiple testing.

Abbreviations: RT, retention time; GDM, gestational diabetes mellitus; DMG, dimethylglycine; TMAO, trimethylamine N-oxide; GPC, sn-glycerol-3-phosphocholine; GABA, gamma-aminobutyric acid; ADMA, Asymmetric dimethylarginine; SAH, S-adenosylhomocysteine.

**Supplementary Table S4. List of lipid metabolites identified in sera obtained from pregnant women in the first and second trimester.**

| **Lipid Species** | **m/z** | **RT** | **Ion Species** | **1st trimester** | | | | | | **2nd trimester** | | | | | |  |
| --- | --- | --- | --- | --- | --- | --- | --- | --- | --- | --- | --- | --- | --- | --- | --- | --- |
|  |  |  |  | **non-GDM (n=118)** | | **GDM  (n=118)** | | ***P*** | ***P_adj_*** | **non-GDM  (n=118)** | | **GDM  (n=118)** | | ***P*** | ***P_adj_*** |  |
| FFA 12:0 | 199.1688 | 1.14 | [M-H]^-^ | 25.18 | (18.91 - 32.81) | 20.56 | (15.79 - 28.07) | <0.001 | 0.005 | 10.98 | (9.01 - 15.46) | 10.49 | (8.46 - 16.2) | 0.569 | 0.298 |  |
| FFA 14:1 | 225.1848 | 1.26 | [M-H]^-^ | 15.11 | (9.49 - 24.19) | 11.05 | (7.4 - 18.61) | 0.006 | 0.022 | 0.67 | (0.19 - 2.42) | 2.16 | (0.96 - 4.06) | <0.001 | <0.001 |  |
| FFA 14:0 | 227.2000 | 1.70 | [M-H]^-^ | 188.97 | (146.75 - 238.77) | 173.37 | (144.5 - 219.57) | 0.118 | 0.180 | 87.17 | (73.64 - 108.95) | 102.16 | (84.08 - 131.03) | <0.001 | 0.001 |  |
| FFA 16:2 | 251.2013 | 1.44 | [M-H]^-^ | 1.8 | (0.83 - 2.68) | 1.44 | (0.4 - 2.62) | 0.106 | 0.168 | 0.16 | (0 - 0.33) | 0.33 | (0 - 0.73) | <0.001 | <0.001 |  |
| FFA 16:1 | 253.2160 | 1.88 | [M-H]^-^ | 643.8 | (441.31 - 852.81) | 589.97 | (383.81 - 781.79) | 0.091 | 0.153 | 127.23 | (82.08 - 169.32) | 208.83 | (126.32 - 302.89) | <0.001 | <0.001 |  |
| FFA 16:0 | 255.2316 | 2.66 | [M-H]^-^ | 12162.22 | (10625.27 - 14264.15) | 11510.4 | (9803.78 - 13155.21) | 0.027 | 0.065 | 8043.64 | (7212.06 - 8900.55) | 7891.79 | (7167.02 - 9355.76) | 0.962 | 0.417 |  |
| FFA 18:3 | 277.2160 | 1.64 | [M-H]^-^ | 298.24 | (210.85 - 416.11) | 292.73 | (195.48 - 389) | 0.659 | 0.493 | 68.32 | (44.63 - 94.47) | 97.9 | (68.64 - 168.07) | <0.001 | <0.001 |  |
| FFA 18:2 | 279.2319 | 2.14 | [M-H]^-^ | 4781.03 | (3527.66 - 5812.83) | 4229.03 | (3031.08 - 5381.29) | 0.022 | 0.056 | 1371.75 | (1050.53 - 1714.31) | 1893.82 | (1349.99 - 2514.28) | <0.001 | <0.001 |  |
| FFA 18:1 | 281.2475 | 2.86 | [M-H]^-^ | 10727.27 | (8030.57 - 12843.86) | 9211.02 | (6698.93 - 12466.71) | 0.054 | 0.107 | 2832.5 | (2014.48 - 3551.94) | 3828.3 | (2880.52 - 5303.83) | <0.001 | <0.001 |  |
| FFA 18:0 | 283.2631 | 3.64 | [M-H]^-^ | 13050.25 | (11321.37 - 15191.13) | 12321.57 | (10825.6 - 13501.6) | 0.007 | 0.022 | 10420.3 | (9639.98 - 11177.34) | 9662.72 | (8612.02 - 10773.59) | 0.002 | 0.004 |  |
| FFA 20:5 | 301.2165 | 1.54 | [M-H]^-^ | 20.62 | (12.41 - 37.44) | 18.92 | (13.35 - 32.29) | 0.630 | 0.482 | 7.75 | (4.82 - 12.98) | 11.81 | (6.64 - 18.62) | 0.001 | 0.004 |  |
| FFA 20:4 | 303.2321 | 2.00 | [M-H]^-^ | 370.35 | (286.67 - 441.2) | 359.7 | (297.85 - 459.02) | 0.796 | 0.540 | 187.5 | (140.41 - 242.17) | 220.97 | (183.26 - 282) | <0.001 | <0.001 |  |
| FFA 20:3 | 305.2479 | 2.49 | [M-H]^-^ | 50.38 | (39.22 - 72.08) | 58.29 | (41.7 - 73) | 0.132 | 0.193 | 21.47 | (13.77 - 27.84) | 29.48 | (19.56 - 44.4) | <0.001 | <0.001 |  |
| FFA 20:2 | 307.2635 | 3.06 | [M-H]^-^ | 50.35 | (38.42 - 63.91) | 49.23 | (36.07 - 64.16) | 0.426 | 0.386 | 12.05 | (7.93 - 18.9) | 21.27 | (13.82 - 32.24) | <0.001 | <0.001 |  |
| FFA 20:1 | 309.2791 | 3.80 | [M-H]^-^ | 50.51 | (35.46 - 69.78) | 45.68 | (31.91 - 69.36) | 0.360 | 0.350 | 13.22 | (9.04 - 18.35) | 22.19 | (14.82 - 32.43) | <0.001 | <0.001 |  |
| FFA 20:0 | 311.2946 | 5.06 | [M-H]^-^ | 12.82 | (10.91 - 16.37) | 11.68 | (9.61 - 14.32) | 0.006 | 0.021 | 10.76 | (9.02 - 12.29) | 10.18 | (8.35 - 11.8) | 0.176 | 0.126 |  |
| FFA 22:6 | 327.2322 | 1.76 | [M-H]^-^ | 292.56 | (217.3 - 390.28) | 289.88 | (204.85 - 381.51) | 0.574 | 0.459 | 105.27 | (81.01 - 141.45) | 138.81 | (104.49 - 192.96) | <0.001 | <0.001 |  |
| FFA 22:5 | 329.2481 | 2.22 | [M-H]^-^ | 41.08 | (31.04 - 60.48) | 45.51 | (27.14 - 64.84) | 0.774 | 0.533 | 9.52 | (5.94 - 14.64) | 15.51 | (10.06 - 21.78) | <0.001 | <0.001 |  |
| FFA 22:4 | 331.2645 | 2.85 | [M-H]^-^ | 33.03 | (26.23 - 43.12) | 34.95 | (28.18 - 43.99) | 0.344 | 0.341 | 13.36 | (10.57 - 16.68) | 17.97 | (13.83 - 23.12) | <0.001 | <0.001 |  |
| FFA 22:1 | 337.3101 | 5.29 | [M-H]^-^ | 0.35 | (0.02 - 0.68) | 0.31 | (0.09 - 0.68) | 0.975 | 0.590 | 0.16 | (0 - 0.39) | 0.27 | (0.05 - 0.63) | 0.004 | 0.008 |  |
| FFA 22:0 | 339.3257 | 7.19 | [M-H]^-^ | 1.19 | (0.74 - 1.68) | 1.07 | (0.74 - 1.56) | 0.384 | 0.364 | 1.35 | (0.85 - 2.34) | 1.25 | (0.79 - 2.15) | 0.744 | 0.356 |  |
| FFA 26:0 | 395.3885 | 13.12 | [M-H]^-^ | 45.47 | (39.74 - 52.77) | 42.09 | (38.65 - 47.31) | 0.016 | 0.045 | 40.66 | (37.21 - 46.07) | 39.64 | (34.98 - 43.61) | 0.088 | 0.075 |  |
| FFA 28:0 | 423.4207 | 13.90 | [M-H]^-^ | 12.91 | (10.15 - 15.14) | 11.92 | (10.33 - 14.19) | 0.161 | 0.219 | 12.02 | (10.48 - 13.84) | 11.33 | (9.36 - 13.42) | 0.040 | 0.044 |  |
| FFA 30:0 | 451.4515 | 14.53 | [M-H]^-^ | 9.13 | (7.7 - 12.01) | 8.47 | (7.17 - 10.62) | 0.042 | 0.088 | 8.66 | (7.31 - 10.15) | 7.76 | (6.46 - 9.65) | 0.012 | 0.019 |  |
| FFA 32:0 | 479.4841 | 15.06 | [M-H]^-^ | 8.95 | (7.76 - 12.73) | 9.47 | (7.37 - 12.22) | 0.688 | 0.504 | 8.85 | (6.84 - 10.52) | 8.51 | (6.57 - 10.04) | 0.246 | 0.162 |  |
| FFA 34:0 | 507.5161 | 15.52 | [M-H]^-^ | 19.58 | (16.61 - 23.11) | 18.17 | (15.72 - 20.25) | 0.002 | 0.009 | 16.45 | (14.97 - 18.04) | 16.07 | (13.02 - 18.35) | 0.050 | 0.050 |  |
| Cer d42:2 (1) | 670.6488 | 13.45 | [M+Na]^+^ | 559.83 | (516.84 - 618.82) | 564.17 | (496.12 - 640.56) | 0.651 | 0.490 | 534.87 | (481.67 - 590.02) | 566.03 | (496.48 - 633.98) | 0.056 | 0.054 |  |
| Cer d42:2 (2) | 670.6530 | 13.55 | [M+Na]^+^ | 302.52 | (265.32 - 357.35) | 313.67 | (260 - 366.32) | 0.906 | 0.572 | 280.13 | (245.93 - 337.92) | 273.93 | (227.54 - 343.88) | 0.288 | 0.182 |  |
| Cer d42:1 | 672.6774 | 13.98 | [M+Na]^+^ | 867.61 | (764.31 - 1008.92) | 898.24 | (801.03 - 999.48) | 0.183 | 0.237 | 898.72 | (792.7 - 1018.38) | 891.62 | (810.94 - 1007.1) | 0.885 | 0.397 |  |
| SM d32:1 | 675.5947 | 4.13 | [M+H]^+^ | 638.61 | (572.65 - 742.86) | 625.52 | (572.52 - 668.15) | 0.189 | 0.243 | 654.86 | (577.29 - 755.88) | 607.78 | (536.38 - 684.56) | <0.001 | 0.003 |  |
| SM d34:2 | 701.6143 | 4.29 | [M+H]^+^ | 1008.26 | (905.39 - 1137.44) | 1009.42 | (927.86 - 1126.86) | 0.575 | 0.459 | 1036.69 | (939.57 - 1120.91) | 973.16 | (874.65 - 1099.9) | 0.015 | 0.022 |  |
| SM d34:1 | 703.6278 | 5.38 | [M+H]^+^ | 34941.55 | (29922.63 - 41288.22) | 35625.12 | (30866.95 - 40746.25) | 0.535 | 0.441 | 38791.53 | (32601.26 - 44708.22) | 35572.96 | (30582.2 - 42873.01) | 0.062 | 0.058 |  |
| SM d36:3 | 727.6265 | 4.59 | [M+H]^+^ | 15.27 | (9.26 - 21.99) | 15.89 | (10.86 - 21.75) | 0.676 | 0.499 | 16.79 | (12.22 - 22.55) | 16.65 | (12.14 - 22.5) | 0.875 | 0.394 |  |
| SM d36:2 | 729.6460 | 5.62 | [M+H]^+^ | 591.63 | (496.65 - 661.24) | 601.85 | (522.49 - 690.97) | 0.311 | 0.322 | 582.16 | (503.59 - 679.49) | 564.63 | (487.3 - 670.56) | 0.609 | 0.312 |  |
| SM d36:1 | 731.6634 | 7.14 | [M+H]^+^ | 1533.85 | (1411.23 - 1761.1) | 1566.78 | (1405.63 - 1900.8) | 0.421 | 0.383 | 1529.88 | (1374.02 - 1725.91) | 1528.6 | (1316.69 - 1727.78) | 0.657 | 0.329 |  |
| SM d38:1 | 759.6969 | 9.50 | [M+H]^+^ | 727.57 | (662.41 - 820.04) | 720.78 | (666.64 - 829.98) | 0.978 | 0.591 | 743.67 | (668.25 - 847.65) | 714.29 | (638.56 - 791.8) | 0.038 | 0.043 |  |
| SM d40:1 | 787.7298 | 12.47 | [M+H]^+^ | 2328.69 | (2088.52 - 2567.1) | 2430.11 | (2174.28 - 2644.09) | 0.073 | 0.133 | 2434.99 | (2155.84 - 2668.65) | 2341.57 | (2165.08 - 2560.31) | 0.193 | 0.135 |  |
| SM d42:3 | 811.7137 | 9.82 | [M+H]^+^ | 3852.1 | (3425.55 - 4320.34) | 3819.65 | (3524.68 - 4277.22) | 0.997 | 0.595 | 3827.44 | (3424.72 - 4268.09) | 3666.98 | (3310.46 - 4112.95) | 0.057 | 0.055 |  |
| SM d42:2 (1) | 813.7486 | 12.81 | [M+H]^+^ | 1308.1 | (1152.29 - 1512.11) | 1259.34 | (1154.36 - 1402.42) | 0.229 | 0.272 | 1347.65 | (1147.14 - 1482.07) | 1178.77 | (1043.7 - 1339.4) | <0.001 | <0.001 |  |
| SM d42:2 (2) | 813.7503 | 12.35 | [M+H]^+^ | 4254.47 | (3490.21 - 5071.9) | 4337.6 | (3780.3 - 5100.96) | 0.293 | 0.312 | 4430.32 | (3779.3 - 5007.51) | 4265.56 | (3544.9 - 4745.24) | 0.373 | 0.219 |  |
| SM d42:1 | 815.7584 | 13.33 | [M+H]^+^ | 2387.66 | (2192.92 - 2586.11) | 2441.06 | (2220.93 - 2704.02) | 0.302 | 0.317 | 2488 | (2135.43 - 2687.47) | 2341.96 | (2103.15 - 2518.78) | 0.014 | 0.021 |  |
| LysoPC 16:0 | 496.3802 | 1.19 | [M+H]^+^ | 11312.73 | (9201.39 - 13348.99) | 10796.67 | (9208.77 - 12431.15) | 0.476 | 0.412 | 11053 | (9572.46 - 12624.92) | 10940.98 | (9081.62 - 12970.67) | 0.923 | 0.407 |  |
| LysoPC 18:2 | 520.3824 | 1.02 | [M+H]^+^ | 849.36 | (697.67 - 1064.48) | 871.85 | (668.69 - 1119.64) | 0.581 | 0.461 | 775.8 | (635.73 - 922.12) | 748.22 | (574.06 - 894.2) | 0.165 | 0.119 |  |
| LysoPC 18:1 | 522.3979 | 1.24 | [M+H]^+^ | 1342.95 | (1091.06 - 1554.98) | 1231.91 | (1010.52 - 1530.55) | 0.333 | 0.335 | 1181.2 | (1002.91 - 1360.57) | 1088.72 | (889.51 - 1310.95) | 0.026 | 0.033 |  |
| LysoPC 18:0 | 524.4144 | 1.62 | [M+H]^+^ | 2917.9 | (2289.93 - 3631.73) | 2827.36 | (2183.95 - 3465.32) | 0.394 | 0.369 | 2418.14 | (2134.07 - 2891.16) | 2393.33 | (1994.29 - 2810.69) | 0.401 | 0.231 |  |
| LysoPC 20:4 | 544.3853 | 0.98 | [M+H]^+^ | 384.82 | (292.11 - 452.56) | 370.56 | (284.75 - 460.96) | 0.735 | 0.520 | 283.8 | (223.54 - 353.93) | 273.85 | (221.86 - 347.66) | 0.768 | 0.364 |  |
| LysoPC 20:2 | 548.4090 | 1.32 | [M+H]^+^ | 35.35 | (30.71 - 42.21) | 34.94 | (29.08 - 39.79) | 0.309 | 0.321 | 31.59 | (25.59 - 38.19) | 29.58 | (26.28 - 36.38) | 0.366 | 0.216 |  |
| LysoPC 22:6 | 568.3869 | 0.94 | [M+H]^+^ | 195.58 | (146.88 - 237.66) | 166.9 | (139.31 - 214.98) | 0.034 | 0.076 | 141.75 | (119.18 - 185.3) | 132.49 | (110.08 - 169.11) | 0.037 | 0.042 |  |
| PC 32:3 | 728.5931 | 3.89 | [M+H]^+^ | 23.57 | (14.56 - 38.18) | 23.83 | (15.66 - 34.3) | 0.890 | 0.568 | 25.94 | (17.9 - 33.05) | 27.54 | (18.8 - 35.68) | 0.535 | 0.285 |  |
| PC 32:2 | 730.5961 | 4.61 | [M+H]^+^ | 517.25 | (429.12 - 610.91) | 540.31 | (422.17 - 629.54) | 0.770 | 0.532 | 608.83 | (515.98 - 739.74) | 621.2 | (493.5 - 756) | 0.741 | 0.355 |  |
| PC 32:1 | 732.6118 | 5.68 | [M+H]^+^ | 2424.79 | (1899.4 - 3063.45) | 2462.89 | (1911.27 - 3304.64) | 0.698 | 0.507 | 2596.25 | (1970.45 - 3334.92) | 2955.1 | (2341.25 - 3691.15) | 0.017 | 0.024 |  |
| PC 32:0 | 734.6270 | 7.22 | [M+H]^+^ | 1970.91 | (1796.26 - 2173.73) | 1954.01 | (1772.69 - 2208.98) | 0.923 | 0.577 | 1988.72 | (1784.77 - 2214.83) | 2016.06 | (1800.31 - 2274.3) | 0.579 | 0.301 |  |
| PC 34:4 | 754.5977 | 4.43 | [M+H]^+^ | 161.46 | (129.18 - 186.33) | 154.71 | (132.24 - 184.53) | 0.740 | 0.522 | 161.11 | (127.97 - 196.07) | 165.15 | (137.39 - 205.39) | 0.238 | 0.159 |  |
| PC 34:1 | 760.6465 | 7.45 | [M+H]^+^ | 37784.09 | (32327.37 - 44993.31) | 38633.94 | (31691.09 - 45885.71) | 0.538 | 0.443 | 41416.9 | (32263.87 - 51752.42) | 43772.98 | (36042.04 - 52576.52) | 0.246 | 0.162 |  |
| PC 36:6 (1) | 778.5990 | 4.76 | [M+H]^+^ | 385.47 | (314.8 - 462.11) | 400.17 | (335.64 - 466.79) | 0.666 | 0.496 | 427.4 | (352.75 - 496.26) | 414.17 | (337.78 - 502.05) | 0.600 | 0.309 |  |
| PC 36:6 (2) | 778.6017 | 5.01 | [M+H]^+^ | 987.32 | (800.92 - 1272.93) | 984.14 | (862.52 - 1192.58) | 0.515 | 0.432 | 1066.12 | (898.74 - 1314.55) | 1138.25 | (939.64 - 1361.86) | 0.241 | 0.160 |  |
| PC 36:6 (3) | 778.6033 | 4.14 | [M+H]^+^ | 141.22 | (113.62 - 163.24) | 130.39 | (102.41 - 154.97) | 0.088 | 0.149 | 142.79 | (125.08 - 168.86) | 136 | (108.65 - 160.65) | 0.038 | 0.042 |  |
| PC 36:4 | 782.6314 | 5.77 | [M+H]^+^ | 41991.38 | (35729.14 - 48915.82) | 44466.72 | (37410.81 - 51474.43) | 0.070 | 0.129 | 45037.74 | (37931.43 - 50037.49) | 46013.21 | (37776.28 - 56618.22) | 0.143 | 0.106 |  |
| PC 36:2 | 786.6643 | 7.95 | [M+H]^+^ | 34422.88 | (30078.97 - 42419.37) | 35990.27 | (30775.49 - 42490.92) | 0.111 | 0.172 | 36697.27 | (30800.05 - 45035.06) | 36679.41 | (30875.53 - 43249.52) | 0.751 | 0.358 |  |
| PC 36:1 | 788.6801 | 9.83 | [M+H]^+^ | 3858.97 | (3313.96 - 4748.22) | 4035.19 | (3423.27 - 4536.04) | 0.748 | 0.525 | 3838.5 | (3330.98 - 4342.95) | 3889.24 | (3356.5 - 4305.8) | 0.887 | 0.398 |  |
| PC 38:6 | 806.6350 | 5.37 | [M+H]^+^ | 70129.94 | (55967.45 - 82878.62) | 66685.64 | (55832.32 - 83303.71) | 0.574 | 0.459 | 76345.62 | (62569 - 88673.27) | 69700.81 | (57717.98 - 83335.23) | 0.020 | 0.026 |  |
| PC 38:5 (1) | 808.6453 | 6.45 | [M+H]^+^ | 5300.22 | (4774.17 - 5859.09) | 5234.35 | (4758.16 - 5841.53) | 0.950 | 0.584 | 5113.37 | (4666.52 - 5732.21) | 5368.43 | (4821.9 - 6054.8) | 0.061 | 0.057 |  |
| PC 38:5 (2) | 808.6495 | 5.88 | [M+H]^+^ | 5235.4 | (4343.46 - 6128.34) | 5180.89 | (4485.6 - 5807.86) | 0.370 | 0.356 | 4943.26 | (4415.58 - 5557.39) | 4716.17 | (4207.24 - 5425.98) | 0.172 | 0.124 |  |
| PC 38:3 | 812.6817 | 8.54 | [M+H]^+^ | 4635.96 | (3837.72 - 5585.41) | 4903.82 | (4116.32 - 5955.69) | 0.115 | 0.177 | 4265.37 | (3693.99 - 4854.38) | 4871.07 | (4218.99 - 5550.49) | <0.001 | <0.001 |  |
| PC 38:2 | 814.6975 | 10.18 | [M+H]^+^ | 343.66 | (299.49 - 380.73) | 343.47 | (305.72 - 403.87) | 0.556 | 0.450 | 350.19 | (311.59 - 390.84) | 343.44 | (305.38 - 403.23) | 0.916 | 0.406 |  |
| PC 40:6 | 834.6689 | 7.05 | [M+H]^+^ | 8398.06 | (6881.16 - 9394.86) | 7775.22 | (6723.71 - 8933.32) | 0.139 | 0.199 | 7322.09 | (6516.24 - 7975.74) | 6621.96 | (5886.02 - 7522.54) | <0.001 | <0.001 |  |
| PC 40:5 | 836.6797 | 7.69 | [M+H]^+^ | 1083.07 | (933.36 - 1348.89) | 1026.89 | (855.45 - 1296.26) | 0.265 | 0.296 | 930.83 | (814.55 - 1095.57) | 891.87 | (779.19 - 1044.2) | 0.133 | 0.101 |  |
| PC 40:4 | 838.6971 | 9.27 | [M+H]^+^ | 379.01 | (274.74 - 480.1) | 375.88 | (292.71 - 474.75) | 0.701 | 0.508 | 332.11 | (283.11 - 453.96) | 342.23 | (275.04 - 437.45) | 0.820 | 0.379 |  |
| LysoPE 16:0 | 452.2790 | 2.10 | [M-H]^-^ | 5.77 | (3.84 - 8.02) | 6.67 | (4.39 - 9.18) | 0.026 | 0.063 | 11.42 | (9.37 - 15.17) | 12.35 | (9.18 - 15.12) | 0.822 | 0.380 |  |
| LysoPE 18:2 | 476.2790 | 1.71 | [M-H]^-^ | 0.45 | (0 - 1.45) | 0.83 | (0 - 1.77) | 0.090 | 0.152 | 1.03 | (0.42 - 2.83) | 0.86 | (0.26 - 1.83) | 0.125 | 0.097 |  |
| LysoPE 18:0 | 480.3102 | 3.05 | [M-H]^-^ | 10.6 | (7.42 - 12.58) | 12.17 | (9.4 - 15.62) | <0.001 | 0.004 | 14.09 | (12.14 - 17.39) | 15.11 | (10.83 - 19.71) | 0.660 | 0.329 |  |
| LysoPE 20:4 | 500.2803 | 1.65 | [M-H]^-^ | 1.17 | (0.45 - 2.43) | 1.55 | (0.71 - 2.56) | 0.061 | 0.117 | 0.36 | (0 - 0.93) | 0.52 | (0 - 1.44) | 0.015 | 0.021 |  |
| PE 32:1 | 688.4934 | 12.75 | [M-H]^-^ | 3.91 | (2.45 - 6.07) | 5 | (3.29 - 7.66) | 0.014 | 0.040 | 8.37 | (5.82 - 13.43) | 10.67 | (7.65 - 14.84) | 0.018 | 0.024 |  |
| PE 34:2 | 714.5087 | 12.95 | [M-H]^-^ | 202.7 | (151.79 - 269.03) | 247.82 | (173.33 - 326.71) | 0.001 | 0.005 | 656.49 | (520.17 - 802.81) | 644.37 | (494.49 - 863.88) | 0.696 | 0.341 |  |
| PE 34:1 | 716.5263 | 13.38 | [M-H]^-^ | 130.67 | (106.78 - 161.87) | 157.02 | (127.23 - 193.8) | <0.001 | <0.001 | 276.46 | (227.85 - 342.81) | 293.23 | (247.77 - 365.68) | 0.092 | 0.077 |  |
| PE 36:4 | 738.5090 | 12.89 | [M-H]^-^ | 306.47 | (243.63 - 372.03) | 356.33 | (281.93 - 443.31) | <0.001 | 0.005 | 619.93 | (503.18 - 754.24) | 721.73 | (574.27 - 875.29) | 0.001 | 0.004 |  |
| PE 36:3 (1) | 740.5254 | 13.13 | [M-H]^-^ | 72.65 | (59.39 - 86.22) | 82.59 | (70.75 - 98.34) | <0.001 | 0.002 | 127.7 | (111.41 - 155.15) | 132.95 | (109.02 - 155.33) | 0.632 | 0.320 |  |
| PE 36:3 (2) | 740.5256 | 13.04 | [M-H]^-^ | 52.17 | (38.75 - 67.75) | 59.95 | (42.64 - 76.57) | 0.042 | 0.089 | 183.83 | (140.03 - 224.25) | 168.27 | (130.18 - 218.99) | 0.279 | 0.178 |  |
| PE 36:1 | 744.5490 | 13.90 | [M-H]^-^ | 271.94 | (240.89 - 311.02) | 296.49 | (255.96 - 346.32) | <0.001 | 0.001 | 395.02 | (351.32 - 431.7) | 408.71 | (355.89 - 459.94) | 0.119 | 0.093 |  |
| PE 38:6 | 762.5089 | 12.75 | [M-H]^-^ | 728.15 | (586.29 - 932.77) | 824.25 | (602.63 - 960.07) | 0.093 | 0.155 | 1316.08 | (1155.97 - 1593.84) | 1427.5 | (1221.33 - 1728.27) | 0.053 | 0.053 |  |
| PE 38:5 | 764.5235 | 12.97 | [M-H]^-^ | 165.46 | (144.61 - 199.87) | 168.03 | (142.09 - 193.13) | 0.762 | 0.529 | 249.08 | (218.51 - 293.68) | 260.17 | (220.31 - 315.33) | 0.166 | 0.120 |  |
| PE 40:7 | 788.5263 | 12.86 | [M-H]^-^ | 85.81 | (69.87 - 102.46) | 90.93 | (73.91 - 111.65) | 0.081 | 0.142 | 148.36 | (129.65 - 180.38) | 167.16 | (138.51 - 191.9) | 0.014 | 0.021 |  |
| PE 40:6 | 790.5410 | 13.37 | [M-H]^-^ | 573.76 | (469.81 - 695.02) | 783.01 | (567.47 - 932.14) | <0.001 | <0.001 | 960.72 | (795.03 - 1138.33) | 1140.29 | (896.08 - 1414.82) | <0.001 | <0.001 |  |
| PE O-40:7 | 774.5462 | 13.60 | [M-H]^-^ | 256.56 | (199.08 - 349.06) | 254.19 | (192.21 - 344.81) | 0.934 | 0.580 | 283.47 | (217.65 - 361.83) | 270.63 | (205.22 - 354.06) | 0.296 | 0.186 |  |
| PI 32:0 | 809.5225 | 8.28 | [M-H]^-^ | 0.33 | (0 - 2.13) | 0.6 | (0 - 2.46) | 0.298 | 0.315 | 10.8 | (4.63 - 24.42) | 9.49 | (3.08 - 19.32) | 0.336 | 0.203 |  |
| PI 34:2 | 833.5220 | 7.07 | [M-H]^-^ | 71.86 | (51.49 - 93.69) | 75.35 | (58.14 - 101.1) | 0.048 | 0.098 | 157.43 | (114.13 - 212.5) | 128.07 | (101.16 - 187.18) | 0.004 | 0.009 |  |
| PI 34:1 | 835.5380 | 8.73 | [M-H]^-^ | 39.7 | (23.2 - 63.78) | 55.79 | (34.12 - 75.58) | <0.001 | 0.002 | 104.51 | (77.43 - 136.57) | 104.34 | (77.31 - 137.82) | 0.706 | 0.344 |  |
| PI 36:4 | 857.5223 | 6.94 | [M-H]^-^ | 68.52 | (49.06 - 100.15) | 80.4 | (59.31 - 110.66) | 0.069 | 0.127 | 109.36 | (89.87 - 158.32) | 100.68 | (79.81 - 145.54) | 0.076 | 0.067 |  |
| PI 36:3 (1) | 859.5384 | 7.75 | [M-H]^-^ | 0.78 | (0 - 4.28) | 2.63 | (0.9 - 5.28) | <0.001 | 0.004 | 9.48 | (5.78 - 17.67) | 8.04 | (3.94 - 13.74) | 0.043 | 0.046 |  |
| PI 36:3 (2) | 859.5388 | 7.40 | [M-H]^-^ | 1.58 | (0.59 - 4.4) | 1.03 | (0.22 - 2.79) | 0.021 | 0.055 | 7.77 | (3.29 - 13.38) | 4.29 | (1.92 - 9.06) | <0.001 | 0.002 |  |
| PI 36:2 (1) | 861.5529 | 9.52 | [M-H]^-^ | 285.55 | (211.27 - 357.44) | 300.74 | (231.58 - 389.58) | 0.153 | 0.211 | 578.7 | (462.97 - 723.44) | 517.09 | (410.85 - 638.09) | 0.008 | 0.015 |  |
| PI 36:2 (2) | 861.5545 | 9.14 | [M-H]^-^ | 0.86 | (0.43 - 2.11) | 0.64 | (0 - 1.93) | 0.131 | 0.192 | 4.71 | (2.32 - 8.06) | 2.14 | (0.92 - 4.85) | <0.001 | <0.001 |  |
| PI 36:1 | 863.5691 | 11.67 | [M-H]^-^ | 18.94 | (12.91 - 28.17) | 22.91 | (15.37 - 34.11) | 0.009 | 0.027 | 54.38 | (45.28 - 69.25) | 49.27 | (38.76 - 63.41) | 0.056 | 0.054 |  |
| PI 38:6 | 881.5237 | 6.59 | [M-H]^-^ | 5.42 | (2.13 - 9.84) | 4.76 | (1.87 - 9.88) | 0.642 | 0.486 | 15.19 | (8.25 - 24.51) | 10.66 | (6.26 - 18.33) | 0.007 | 0.013 |  |
| PI 38:5 | 883.5381 | 7.19 | [M-H]^-^ | 1.27 | (0.42 - 3.11) | 1.09 | (0.43 - 3.11) | 0.933 | 0.579 | 3.44 | (1.77 - 6.63) | 3.1 | (1.27 - 6.26) | 0.129 | 0.099 |  |
| PI 38:4 | 885.5526 | 9.37 | [M-H]^-^ | 1114.51 | (907.95 - 1322.73) | 1200.36 | (976.62 - 1346.79) | 0.065 | 0.122 | 1321.18 | (1128.67 - 1614.33) | 1275.03 | (1086.35 - 1548.15) | 0.457 | 0.254 |  |
| PI 38:3 | 887.5691 | 10.43 | [M-H]^-^ | 60.26 | (47.84 - 78.36) | 73.67 | (57.3 - 96.06) | <0.001 | 0.005 | 106.16 | (90.67 - 135.72) | 109.15 | (84.75 - 132.17) | 0.398 | 0.229 |  |
| PI 40:6 | 909.5535 | 8.89 | [M-H]^-^ | 36.43 | (55.69 - 95.69) | 36.07 | (27.32 - 47.45) | 0.971 | 0.589 | 62.17 | (48.31 - 78.17) | 57.05 | (42.2 - 75.89) | 0.106 | 0.086 |  |
| DAG 32:2 | 587.5055 | 7.71 | [M+Na]^+^ | 39.06 | (29.83 - 47.12) | 40.82 | (26.54 - 50.66) | 0.854 | 0.558 | 42.94 | (35.65 - 50.2) | 43.68 | (36.93 - 53.37) | 0.434 | 0.244 |  |
| DAG 34:2 | 615.5398 | 10.24 | [M+Na]^+^ | 592.06 | (542.09 - 654) | 607.39 | (544.35 - 670.45) | 0.442 | 0.395 | 626.53 | (571.41 - 682.25) | 622.84 | (560.23 - 739.94) | 0.409 | 0.234 |  |
| DAG 34:1 | 617.5570 | 12.72 | [M+Na]^+^ | 730.37 | (667.73 - 791.13) | 763.86 | (691.59 - 827.02) | 0.041 | 0.086 | 735.09 | (674.3 - 821.77) | 812.85 | (713 - 892.35) | <0.001 | <0.001 |  |
| DAG 36:4 | 639.5560 | 8.42 | [M+Na]^+^ | 359.07 | (286.42 - 433.02) | 359.95 | (290.64 - 448.74) | 0.967 | 0.588 | 385.71 | (285.51 - 483.26) | 361.84 | (297.19 - 450.47) | 0.422 | 0.239 |  |
| DAG 36:3 | 641.5646 | 10.50 | [M+Na]^+^ | 1202.17 | (1055.41 - 1404.3) | 1256.27 | (1022.53 - 1455.08) | 0.397 | 0.370 | 1278.5 | (1030.12 - 1480.06) | 1269.41 | (1114.54 - 1474.71) | 0.708 | 0.345 |  |
| DAG 38:1 | 673.6235 | 15.37 | [M+Na]^+^ | 224.79 | (192.81 - 260.97) | 222.95 | (194.62 - 258.2) | 0.974 | 0.590 | 207.84 | (182.6 - 231.43) | 194.31 | (170.36 - 219.51) | 0.048 | 0.049 |  |
| TAG 40:2 | 713.6258 | 13.66 | [M+Na]^+^ | 296.06 | (218.17 - 374.62) | 291.02 | (236.82 - 393.71) | 0.375 | 0.359 | 278.38 | (219.86 - 367.42) | 310.32 | (226.85 - 398.63) | 0.197 | 0.137 |  |
| TAG 40:0 | 717.6419 | 14.39 | [M+Na]^+^ | 1024.13 | (910.13 - 1162.08) | 974.12 | (882.63 - 1176.85) | 0.451 | 0.399 | 948.68 | (849.38 - 1098.27) | 914.65 | (788.44 - 1031.77) | 0.049 | 0.050 |  |
| TAG 42:3 | 739.6357 | 13.85 | [M+Na]^+^ | 116.83 | (81.87 - 145.44) | 119.07 | (91.57 - 153.62) | 0.298 | 0.315 | 117.79 | (91.38 - 139.27) | 127.21 | (93.93 - 154.33) | 0.073 | 0.065 |  |
| TAG 42:2 | 741.6588 | 14.06 | [M+Na]^+^ | 151.33 | (112.27 - 218.65) | 182.39 | (134.63 - 237.08) | 0.050 | 0.101 | 196.74 | (153.21 - 289.24) | 217.02 | (164.38 - 284.68) | 0.131 | 0.100 |  |
| TAG 42:1 | 743.6716 | 14.40 | [M+Na]^+^ | 849.56 | (729.52 - 987.25) | 860.36 | (737.49 - 969.71) | 0.693 | 0.506 | 877.69 | (693.04 - 1056.82) | 813.58 | (722.01 - 1043.22) | 0.658 | 0.329 |  |
| TAG 44:2 | 769.6926 | 14.45 | [M+Na]^+^ | 414.83 | (356.64 - 547.24) | 444.24 | (367.34 - 567.39) | 0.318 | 0.326 | 489.68 | (391.71 - 748.17) | 501.11 | (415.4 - 680.56) | 0.779 | 0.367 |  |
| TAG 44:1 | 771.7084 | 14.77 | [M+Na]^+^ | 3309.28 | (2889.85 - 3830.42) | 3320.65 | (2892.32 - 3862.14) | 0.956 | 0.585 | 3353.61 | (2695.39 - 3989.07) | 3148.01 | (2771.33 - 3972.48) | 0.640 | 0.323 |  |
| TAG 46:3 | 795.7077 | 14.48 | [M+Na]^+^ | 418.21 | (302.58 - 541.21) | 377.86 | (302.08 - 552.74) | 0.806 | 0.543 | 494.15 | (370.44 - 728.43) | 488.77 | (371.93 - 677.83) | 0.970 | 0.419 |  |
| TAG 46:2 | 797.7218 | 14.81 | [M+Na]^+^ | 1716.64 | (1371.63 - 2294.05) | 1722.02 | (1408.66 - 2294.97) | 0.529 | 0.438 | 1978.26 | (1564.28 - 2985.23) | 2114.31 | (1734.63 - 2843.4) | 0.570 | 0.298 |  |
| TAG 46:1 | 799.7402 | 15.11 | [M+Na]^+^ | 3619.78 | (3023.93 - 4334.63) | 3804.34 | (3136.4 - 4402.06) | 0.376 | 0.359 | 3781.06 | (3243.93 - 5245.35) | 4025.37 | (3420.99 - 5097.86) | 0.255 | 0.167 |  |
| TAG 46:0 | 801.7489 | 15.42 | [M+Na]^+^ | 4018.65 | (3628.91 - 4698.38) | 3973.19 | (3510.99 - 4523.14) | 0.430 | 0.388 | 3664.87 | (3241.02 - 4381.2) | 3553.12 | (3105.57 - 3976.86) | 0.109 | 0.088 |  |
| TAG 48:0 | 829.7893 | 15.73 | [M+Na]^+^ | 2598.22 | (2252.79 - 3064.25) | 2629.87 | (2341.53 - 3004.94) | 0.740 | 0.522 | 2444.24 | (2055.3 - 2773.06) | 2419.78 | (2033.82 - 2739.01) | 0.438 | 0.246 |  |
| TAG 50:3 | 851.7755 | 15.16 | [M+Na]^+^ | 16424.95 | (14267.16 - 18475.15) | 16578.87 | (13911.45 - 19138.63) | 0.297 | 0.314 | 17339.86 | (15368.1 - 20496.4) | 17938.71 | (15563.81 - 21311.29) | 0.231 | 0.155 |  |
| TAG 50:2 | 853.7934 | 15.43 | [M+Na]^+^ | 16197.83 | (13633.39 - 19966.86) | 17478.21 | (14330.38 - 21528.76) | 0.075 | 0.134 | 18079.94 | (15045.2 - 23544.06) | 21102.02 | (16725.15 - 25264.12) | 0.012 | 0.019 |  |
| TAG 50:1 | 855.8088 | 15.72 | [M+Na]^+^ | 10708.12 | (8241.28 - 14246.36) | 12574.67 | (9701.85 - 16109.12) | 0.005 | 0.019 | 13823.94 | (9818.99 - 18152.72) | 17282.69 | (12244.74 - 21158.06) | 0.002 | 0.005 |  |
| TAG 50:0 | 857.8211 | 16.01 | [M+Na]^+^ | 2219.92 | (1990.02 - 2497) | 2310.81 | (2111.72 - 2624.32) | 0.021 | 0.054 | 2248 | (2005.86 - 2442.27) | 2157.7 | (1912.78 - 2456.65) | 0.304 | 0.189 |  |
| TAG 52:3 | 879.8069 | 15.47 | [M+Na]^+^ | 55246.14 | (46798.81 - 59967.21) | 56169.88 | (49590.59 - 63457.8) | 0.186 | 0.240 | 58440.68 | (49962.63 - 64511.36) | 56606.7 | (50162.17 - 63704.17) | 0.476 | 0.262 |  |
| TAG 52:2 | 881.8250 | 15.72 | [M+Na]^+^ | 36427.23 | (31809.88 - 41772.09) | 38400.56 | (32045.55 - 45092.13) | 0.022 | 0.056 | 38618.62 | (33133.5 - 43919.06) | 39734.24 | (35201.57 - 47007.38) | 0.211 | 0.145 |  |
| TAG 52:1 | 883.8422 | 16.00 | [M+Na]^+^ | 4199.06 | (3270.77 - 5957.9) | 5507.16 | (3960.57 - 7239.55) | <0.001 | 0.002 | 5942.58 | (4287.76 - 7748.48) | 7064.51 | (5135.49 - 8654.15) | 0.006 | 0.011 |  |
| TAG 52:0 | 885.8521 | 16.26 | [M+Na]^+^ | 1056.3 | (975.88 - 1193.02) | 1099.76 | (981.5 - 1222.41) | 0.408 | 0.376 | 1008.71 | (917.32 - 1147.16) | 981.65 | (860.04 - 1099.27) | 0.074 | 0.066 |  |
| TAG 54:5 | 903.8117 | 15.21 | [M+Na]^+^ | 14549.11 | (12244.53 - 16737.61) | 14573.61 | (12234.45 - 15972.99) | 0.619 | 0.477 | 15436.7 | (12963.34 - 18602.22) | 14030.65 | (12423.22 - 16709.16) | 0.025 | 0.031 |  |
| TAG 54:4 | 905.8293 | 15.49 | [M+Na]^+^ | 12210.27 | (10362.17 - 14293.05) | 12373.07 | (10484.5 - 14360.32) | 0.909 | 0.573 | 13611.47 | (11415.86 - 15804.74) | 12339.92 | (10859.05 - 14231.24) | 0.063 | 0.058 |  |
| TAG 54:3 | 907.8450 | 15.76 | [M+Na]^+^ | 9764.98 | (8223.1 - 11529.68) | 10089.32 | (8315.67 - 11649.89) | 0.456 | 0.402 | 10789.7 | (9072.81 - 12671.2) | 10018.3 | (8793.54 - 11900.89) | 0.055 | 0.054 |  |
| TAG 54:2 | 909.8603 | 15.99 | [M+Na]^+^ | 4039.98 | (3321.59 - 4864.93) | 4564.6 | (3660.12 - 5486.67) | 0.002 | 0.007 | 5042.61 | (4031.77 - 6533.69) | 5178.37 | (4225.08 - 6708.25) | 0.375 | 0.220 |  |
| TAG 54:1 | 911.8718 | 16.25 | [M+Na]^+^ | 620.91 | (546.86 - 798.23) | 724.73 | (607.25 - 892.71) | <0.001 | 0.003 | 769.01 | (638.99 - 993.14) | 843.62 | (670.07 - 1031.13) | 0.112 | 0.089 |  |
| TAG 54:0 | 913.8839 | 16.50 | [M+Na]^+^ | 627.71 | (558.45 - 711.43) | 617.3 | (562.07 - 695.58) | 0.722 | 0.516 | 560.22 | (512.74 - 637.29) | 555.57 | (468.18 - 610.17) | 0.082 | 0.071 |  |
| TAG 56:9 | 923.7828 | 14.70 | [M+Na]^+^ | 2048.37 | (1747.09 - 2561.91) | 1949.31 | (1667.18 - 2457.85) | 0.365 | 0.353 | 1898.02 | (1538.52 - 2341.67) | 2093.45 | (1807.05 - 2675.95) | 0.013 | 0.020 |  |
| TAG 56:8 | 925.8004 | 14.97 | [M+Na]^+^ | 8520.27 | (7099.27 - 9940.41) | 7925.54 | (6537.41 - 9874.43) | 0.302 | 0.317 | 8027.61 | (6529.27 - 9496.71) | 8743.31 | (6963.95 - 10846.15) | 0.008 | 0.014 |  |
| TAG 56:7 (1) | 927.8157 | 15.09 | [M+Na]^+^ | 3272.43 | (2907.29 - 3892.33) | 3281.25 | (2745.77 - 3727.25) | 0.517 | 0.433 | 3074.39 | (2518.27 - 3552.67) | 3068.02 | (2681.33 - 3431.66) | 0.843 | 0.385 |  |
| TAG 56:7 (2) | 927.8191 | 15.24 | [M+Na]^+^ | 9714.38 | (8091.1 - 11034.55) | 9431.11 | (7898.46 - 10997.36) | 0.691 | 0.505 | 9161.45 | (7555.84 - 10621.27) | 9634.14 | (8322.94 - 11556.06) | 0.008 | 0.015 |  |
| TAG 56:5 (1) | 931.8446 | 15.58 | [M+Na]^+^ | 1489.28 | (1266.91 - 1766.54) | 1563.6 | (1382.57 - 1837.03) | 0.098 | 0.160 | 1658.53 | (1441.28 - 1921.26) | 1773.29 | (1503.08 - 2048.53) | 0.031 | 0.037 |  |
| TAG 56:5 (2) | 931.8480 | 15.66 | [M+Na]^+^ | 1345.02 | (1147.32 - 1608.8) | 1456.89 | (1223.74 - 1711.25) | 0.041 | 0.087 | 1371.22 | (1199.48 - 1620.47) | 1435.21 | (1235.98 - 1661.11) | 0.242 | 0.161 |  |
| TAG 56:4 | 933.8594 | 15.80 | [M+Na]^+^ | 899.21 | (791.05 - 1016.49) | 912.26 | (803.39 - 998.35) | 0.480 | 0.415 | 994.55 | (911.81 - 1139.95) | 990.06 | (898.9 - 1087.09) | 0.610 | 0.312 |  |
| TAG 56:3 | 935.8744 | 15.99 | [M+Na]^+^ | 635.21 | (538.36 - 741.54) | 656.05 | (565.76 - 762.35) | 0.224 | 0.269 | 775.7 | (668.11 - 951.69) | 770.62 | (652.46 - 913.22) | 0.418 | 0.238 |  |
| TAG 56:0 | 941.9166 | 16.72 | [M+Na]^+^ | 415.55 | (370.74 - 460.49) | 399.71 | (364.8 - 456.95) | 0.309 | 0.321 | 366.12 | (322.3 - 408.81) | 351.45 | (299.99 - 398.58) | 0.095 | 0.079 |  |
| TAG 58:11 | 947.7991 | 14.62 | [M+Na]^+^ | 137.53 | (88.21 - 190.5) | 132.33 | (90.54 - 219.32) | 0.937 | 0.580 | 120.9 | (79.96 - 192.58) | 149.48 | (99.22 - 234.92) | 0.011 | 0.018 |  |
| TAG 58:10 (1) | 949.7948 | 14.69 | [M+Na]^+^ | 1697.76 | (1425.04 - 2078.89) | 1592.89 | (1374.85 - 1923.99) | 0.110 | 0.172 | 1535.15 | (1258.61 - 1844.91) | 1648.29 | (1329.29 - 1854.22) | 0.228 | 0.153 |  |
| TAG 58:10 (2) | 949.8008 | 14.87 | [M+Na]^+^ | 345.56 | (237.17 - 441.95) | 303.17 | (246.02 - 430.61) | 0.432 | 0.389 | 300.52 | (228.26 - 391.82) | 346.71 | (258.25 - 467.41) | 0.008 | 0.014 |  |
| TAG 58:9 | 951.8127 | 14.98 | [M+Na]^+^ | 3115.36 | (2624.62 - 3446.91) | 2917.28 | (2374.5 - 3348.36) | 0.147 | 0.206 | 2852.87 | (2242.98 - 3276.99) | 2895.19 | (2529.17 - 3298.37) | 0.139 | 0.104 |  |
| TAG 58:8 | 953.8330 | 15.26 | [M+Na]^+^ | 3195.05 | (2756.52 - 3561.7) | 2949.92 | (2566.09 - 3462.48) | 0.124 | 0.185 | 3161.03 | (2618.4 - 3650.17) | 3182.99 | (2831.4 - 3697.14) | 0.509 | 0.275 |  |
| TAG 58:6 | 957.8617 | 15.59 | [M+Na]^+^ | 1143.75 | (994.72 - 1412.9) | 1141.36 | (1020.7 - 1327.04) | 0.915 | 0.574 | 1355.46 | (1176.81 - 1583.83) | 1346.52 | (1216.47 - 1639.78) | 0.591 | 0.305 |  |
| TAG 58:5 | 959.8790 | 15.81 | [M+Na]^+^ | 595.21 | (525.99 - 667.42) | 587.35 | (529.09 - 662.31) | 0.841 | 0.554 | 635.73 | (586.06 - 721.89) | 642.33 | (583.77 - 709.63) | 0.830 | 0.382 |  |
| TAG 58:4 | 961.8889 | 16.00 | [M+Na]^+^ | 185.58 | (155.41 - 241.96) | 201.09 | (151.14 - 254.14) | 0.482 | 0.416 | 227.88 | (195.35 - 257.7) | 220.52 | (196.38 - 253) | 0.306 | 0.190 |  |
| TAG 58:3 | 963.9022 | 16.24 | [M+Na]^+^ | 142.95 | (110.92 - 199.53) | 148.98 | (115.74 - 203.93) | 0.235 | 0.277 | 193.77 | (162.64 - 237.36) | 190.33 | (158.09 - 232.64) | 0.637 | 0.322 |  |
| TAG 60:13 | 971.8028 | 14.32 | [M+Na]^+^ | 184.76 | (122.5 - 283.69) | 186.36 | (131.66 - 280.27) | 0.646 | 0.488 | 152.59 | (91.55 - 259.23) | 198.62 | (138.46 - 354.15) | 0.006 | 0.012 |  |
| TAG 60:12 (1) | 973.8048 | 14.73 | [M+Na]^+^ | 592.89 | (391.7 - 829.5) | 486.32 | (369.57 - 739.26) | 0.099 | 0.161 | 519.19 | (316.45 - 704.67) | 573.59 | (426.51 - 810.97) | 0.023 | 0.029 |  |
| TAG 60:12 (2) | 973.8101 | 14.58 | [M+Na]^+^ | 470.46 | (328.55 - 654.09) | 481.8 | (354.18 - 617.2) | 0.688 | 0.504 | 385.75 | (270.01 - 608.37) | 480.05 | (371.18 - 633.97) | 0.002 | 0.004 |  |
| TAG 60:11 (1) | 975.8180 | 14.87 | [M+Na]^+^ | 524.21 | (403.36 - 657.98) | 492.5 | (375.77 - 584.43) | 0.148 | 0.207 | 424.79 | (329.65 - 559.92) | 506.47 | (389.18 - 616.8) | 0.006 | 0.011 |  |
| TAG 60:11 (2) | 975.8231 | 14.98 | [M+Na]^+^ | 59.72 | (39.26 - 86.49) | 55.58 | (35.85 - 74.63) | 0.315 | 0.324 | 58.71 | (34.9 - 77.26) | 62.67 | (48.94 - 79.63) | 0.102 | 0.083 |  |
| TAG 60:10 (1) | 977.8321 | 15.09 | [M+Na]^+^ | 389.81 | (330.83 - 475.13) | 380.63 | (292.3 - 450.31) | 0.148 | 0.208 | 361.16 | (291.81 - 454.4) | 384.83 | (327.22 - 447.51) | 0.117 | 0.092 |  |
| TAG 60:10 (2) | 977.8366 | 15.19 | [M+Na]^+^ | 138.86 | (105.49 - 170.03) | 126.18 | (101.91 - 167.39) | 0.255 | 0.289 | 111.89 | (79.94 - 150.57) | 128.33 | (92.94 - 161.27) | 0.091 | 0.077 |  |
| TAG 60:9 | 979.8513 | 15.31 | [M+Na]^+^ | 148.57 | (128.21 - 185.05) | 144.89 | (123.22 - 169.73) | 0.230 | 0.273 | 141.15 | (115.01 - 170.79) | 146.31 | (124.95 - 174.22) | 0.103 | 0.084 |  |
| TAG 60:8 | 981.8607 | 15.34 | [M+Na]^+^ | 249.08 | (185.48 - 303.83) | 231.59 | (172.95 - 305.69) | 0.229 | 0.272 | 275.34 | (212.07 - 318) | 254.53 | (207.04 - 320.07) | 0.513 | 0.276 |  |
| TAG 60:7 | 983.8667 | 15.63 | [M+Na]^+^ | 262.46 | (201.18 - 325.94) | 249.66 | (195.15 - 317.73) | 0.374 | 0.358 | 295.34 | (244.24 - 338.58) | 291.74 | (245.81 - 336.43) | 0.544 | 0.288 |  |
| TAG 60:6 | 985.8917 | 15.83 | [M+Na]^+^ | 145.61 | (102.38 - 193.14) | 140.65 | (101.93 - 194.4) | 0.973 | 0.589 | 173.08 | (147.57 - 209.17) | 170.96 | (138.03 - 205.03) | 0.536 | 0.285 |  |
| TAG 62:14 | 997.8049 | 14.44 | [M+Na]^+^ | 137.35 | (74.78 - 234.9) | 115.23 | (63.02 - 195.29) | 0.185 | 0.239 | 99.96 | (49.1 - 199.14) | 131.61 | (73.98 - 198.62) | 0.022 | 0.029 |  |
| TAG 62:13 | 999.8208 | 14.74 | [M+Na]^+^ | 257.29 | (172.3 - 371.87) | 228.78 | (153.96 - 345.9) | 0.286 | 0.308 | 209.66 | (120.16 - 318.07) | 250.05 | (165.25 - 345.54) | 0.014 | 0.021 |  |
| TAG 62:12 | 1001.8336 | 14.94 | [M+Na]^+^ | 59.46 | (35.35 - 86.98) | 56.71 | (33.98 - 78.07) | 0.281 | 0.305 | 54.46 | (34.38 - 77.57) | 61.5 | (44.44 - 82.81) | 0.068 | 0.062 |  |

The values are expressed as the median/1,000 (interquartile range).

*P*-values were obtained using the Ranked ANCOVA test with adjustments for maternal age, pre-pregnancy body mass index, nulliparity, and family history of diabetes.

*P_adj_*-values were calculated using the Benjamini–Hochberg method to adjust multiple testing.

Abbreviations: RT, retention time; GDM, gestational diabetes mellitus; FFA, free fatty acid; Cer, ceramide; SM, sphingomyelin; lysoPC, lysophosphatidylcholine; PC, phosphatidylcholine; lysoPE, lysophosphatidylethanolamine; PE, phosphatidylethanolamine; PI, phosphatidylinositol; DAG, diacylglycerol; TAG, triacylglycerol.

**Supplementary Table S5. Mediation analysis for circulating metabolites and GDM via MASLD.**

| **Metabolites** | **Total effect** | | **Direct effect** | | **Indirect effect** | | **PM (%)** |
| --- | --- | --- | --- | --- | --- | --- | --- |
|  | **β (95% CI)** | ***P*** | **β (95% CI)** | ***P*** | **β (95% CI)** | ***P*** |  |
| GCA | 0.18 (0.06-0.30) | 0.000 | 0.14 (0.03-0.26) | 0.014 | 0.04 (0.00-0.09) | 0.028 | 21.6 |
| GCDCA | 0.14 (0.02-0.26) | 0.016 | 0.12 (0.01-0.24) | 0.034 | 0.02 (-0.02-0.06) | 0.312 | 13.0 |
| TCA | 0.22 (0.10-0.34) | 0.000 | 0.19 (0.07-0.30) | 0.000 | 0.03 (-0.00-0.08) | 0.072 | 14.3 |
| TDCA | 0.17 (0.04-0.28) | 0.006 | 0.15 (0.04-0.26) | 0.010 | 0.01 (-0.02-0.05) | 0.450 | 7.7 |
| TCDCA | 0.21 (0.09-0.33) | 0.000 | 0.20 (0.08-0.31) | 0.000 | 0.01 (-0.02-0.05) | 0.456 | 6.2 |
| Acetyl carnitine | -0.13 (-0.25--0.01) | 0.032 | -0.16 (-0.27--0.04) | 0.006 | 0.03 (-0.01-0.07) | 0.192 | NA |
| Butanoyl carnitine | 0.16 (0.03-0.28) | 0.014 | 0.11 (0.00-0.24) | 0.066 | 0.04 (0.00-0.09) | 0.028 | 25.9 |
| Betaine | -0.17 (-0.30--0.05) | 0.002 | -0.17 (-0.28--0.05) | 0.006 | -0.01 (-0.05-0.03) | 0.710 | 3.1 |
| Guanine | 0.18 (0.06-0.31) | 0.002 | 0.16 (0.04-0.28) | 0.008 | 0.03 (-0.01-0.07) | 0.168 | 13.9 |
| Inosine | 0.24 (0.12-0.35) | 0.000 | 0.21 (0.10-0.33) | <0.001 | 0.03 (0.00-0.07) | 0.082 | 11.4 |
| Uric acid | 0.16 (0.04-0.27) | 0.008 | 0.12 (0.00-0.23) | 0.052 | 0.04 (0.00-0.09) | 0.006 | 24.6 |
| Cystathionine | 0.22 (0.10-0.34) | 0.000 | 0.20 (0.08-0.32) | 0.000 | 0.02 (-0.01-0.06) | 0.236 | 9.1 |
| 3-Hydroxybutyrate | -0.13 (-0.25--0.01) | 0.034 | -0.14 (-0.25--0.02) | 0.018 | 0.01 (-0.03-0.05) | 0.636 | NA |
| Acetoacetate | -0.19 (-0.30--0.07) | 0.002 | -0.2 (-0.31--0.09) | 0.000 | 0.01 (-0.03-0.05) | 0.608 | NA |
| Leucine | 0.14 (0.01-0.27) | 0.026 | 0.11 (-0.02-0.24) | 0.102 | 0.03 (0.00-0.08) | 0.078 | 22.0 |
| Isoleucine | 0.16 (0.03-0.28) | 0.010 | 0.14 (0.01-0.27) | 0.028 | 0.02 (-0.02-0.06) | 0.320 | 11.7 |
| Valine | 0.20 (0.08-0.32) | 0.000 | 0.16 (0.04-0.29) | 0.004 | 0.04 (0.00-0.08) | 0.056 | 17.1 |
| Glutamine | -0.13 (-0.25--0.01) | 0.036 | -0.11 (-0.23-0.01) | 0.064 | -0.02 (-0.07-0.02) | 0.300 | 14.3 |
| Tyrosine | 0.19 (0.07-0.32) | 0.002 | 0.16 (0.04-0.29) | 0.006 | 0.03 (0.00-0.08) | 0.086 | 15.0 |
| Glycine | -0.14 (-0.26--0.01) | 0.040 | -0.10 (-0.22-0.02) | 0.100 | -0.03 (-0.08-0.00) | 0.096 | 20.8 |
| Serine | -0.14 (-0.26--0.01) | 0.030 | -0.11 (-0.22-0.01) | 0.076 | -0.03 (-0.08-0.01) | 0.120 | 19.9 |
| Hydroxy-proline | 0.18 (0.06-0.30) | 0.002 | 0.14 (0.02-0.26) | 0.014 | 0.04 (0.00-0.08) | 0.034 | 19.8 |
| Proline | 0.28 (0.16-0.40) | 0.000 | 0.28 (0.16-0.39) | 0.000 | 0.00 (-0.03-0.04) | 0.808 | 1.5 |
| Arginine | 0.29 (0.17-0.41) | 0.000 | 0.26 (0.14-0.39) | 0.000 | 0.03 (0.00-0.07) | 0.074 | 9.7 |
| Alanine | 0.27 (0.15-0.39) | 0.000 | 0.24 (0.12-0.36) | 0.000 | 0.04 (0.00-0.08) | 0.038 | 11.9 |
| Cysteine | 0.16 (0.03-0.27) | 0.008 | 0.14 (0.03-0.26) | 0.010 | 0.01 (-0.02-0.05) | 0.534 | 7.3 |
| Threonine | 0.23 (0.11-0.35) | 0.000 | 0.22 (0.10-0.34) | 0.000 | 0.01 (-0.03-0.05) | 0.662 | 3.6 |
| Kynurenine | 0.13 (0.01-0.25) | 0.032 | 0.09 (-0.03-0.21) | 0.152 | 0.04 (0.01-0.10) | 0.006 | 31.9 |
| FFA 12:0 | -0.20 (-0.32--0.08) | 0.002 | -0.18 (-0.30--0.07) | 0.002 | -0.02 (-0.07-0.02) | 0.294 | 9.0 |
| FFA 14:1 | -0.18 (-0.30--0.06) | 0.002 | -0.17 (-0.29--0.06) | 0.004 | -0.01 (-0.05-0.03) | 0.558 | 5.2 |
| FFA14:0 | -0.12 (-0.25--0.01) | 0.038 | -0.14 (-0.25--0.02) | 0.022 | 0.01 (-0.03-0.05) | 0.562 | NA |
| FFA 16:1 | -0.15 (-0.27--0.03) | 0.016 | -0.16 (-0.27--0.04) | 0.006 | 0.01 (-0.03-0.05) | 0.566 | NA |
| FFA 18:2 | -0.16 (-0.28--0.04) | 0.006 | -0.15 (-0.26--0.03) | 0.008 | -0.01 (-0.05-0.03) | 0.614 | 5.1 |
| FFA 18:1 | -0.17 (-0.29--0.05) | 0.004 | -0.18 (-0.29--0.07) | 0.000 | 0.02 (-0.02-0.06) | 0.428 | NA |
| FFA 20:1 | -0.15 (-0.27--0.03) | 0.012 | -0.16 (-0.27--0.05) | 0.006 | 0.01 (-0.03-0.05) | 0.536 | NA |
| FFA 20:0 | -0.14 (-0.26--0.01) | 0.028 | -0.11 (-0.22-0.00) | 0.062 | -0.03 (-0.08-0.01) | 0.100 | 21.4 |
| LysoPE 16:0 | 0.16 (0.04-0.28) | 0.008 | 0.15 (0.03-0.26) | 0.008 | 0.01 (-0.02-0.05) | 0.406 | 8.3 |
| LysoPE 18:0 | 0.15 (0.03-0.26) | 0.008 | 0.14 (0.02-0.25) | 0.016 | 0.01 (-0.03-0.05) | 0.560 | 6.8 |
| LysoPE 20:4 | 0.15 (0.02-0.27) | 0.012 | 0.12 (0.00-0.24) | 0.058 | 0.03 (0.00-0.07) | 0.108 | 19.1 |
| PE 34:2 | 0.22 (0.10-0.34) | 0.000 | 0.21 (0.10-0.33) | 0.000 | 0.01 (-0.02-0.05) | 0.492 | 5.0 |
| PE 34:1 | 0.20 (0.07-0.32) | 0.002 | 0.17 (0.04-0.28) | 0.006 | 0.03 (0.00-0.07) | 0.108 | 14.7 |
| PE 36:4 | 0.20 (0.08-0.32) | 0.002 | 0.17 (0.05-0.29) | 0.002 | 0.02 (-0.01-0.06) | 0.192 | 11.6 |
| PE 36:3 (1) | 0.23 (0.11-0.34) | 0.000 | 0.21 (0.10-0.33) | 0.000 | 0.02 (-0.02-0.06) | 0.344 | 7.0 |
| PE 36:3 (2) | 0.14 (0.02-0.26) | 0.014 | 0.15 (0.04-0.26) | 0.008 | -0.01 (-0.05-0.03) | 0.736 | NA |
| PE 36:1 | 0.17 (0.05-0.29) | 0.006 | 0.14 (0.03-0.26) | 0.018 | 0.03 (-0.01-0.07) | 0.114 | 16.8 |
| PE 40:6 | 0.28 (0.17-0.40) | 0.000 | 0.24 (0.12-0.36) | 0.000 | 0.04 (0.01-0.09) | 0.010 | 14.5 |
| PI 34:1 | 0.19 (0.06-0.32) | 0.004 | 0.16 (0.04-0.29) | 0.006 | 0.02 (-0.01-0.06) | 0.232 | 11.2 |
| PI 36:3 (1) | 0.18 (0.06-0.30) | 0.002 | 0.16 (0.04-0.28) | 0.004 | 0.02 (-0.02-0.06) | 0.338 | 9.2 |
| PI 36:1 | 0.14 (0.02-0.26) | 0.018 | 0.12 (0.00-0.23) | 0.040 | 0.03 (-0.01-0.07) | 0.212 | 16.2 |
| PI 38:3 | 0.14 (0.02-0.26) | 0.024 | 0.10 (-0.01-0.22) | 0.094 | 0.03 (0.00-0.08) | 0.098 | 22.7 |
| LysoPC 22:6 | -0.12 (-0.25-0.00) | 0.044 | -0.12 (-0.24--0.01) | 0.034 | 0.00 (-0.04-0.04) | 1.000 | 0.6 |
| SM d40:1 | 0.17 (0.05-0.29) | 0.006 | 0.14 (0.03-0.26) | 0.014 | 0.03 (0.00-0.07) | 0.082 | 16.8 |
| TAG 52:1 | 0.20 (0.08-0.32) | 0.000 | 0.18 (0.06-0.29) | 0.002 | 0.03 (-0.01-0.07) | 0.144 | 12.6 |
| TAG 54:2 | 0.18 (0.06-0.30) | 0.002 | 0.17 (0.05-0.28) | 0.000 | 0.02 (-0.02-0.06) | 0.316 | 9.9 |
| TAG 54:1 | 0.22 (0.10-0.34) | 0.000 | 0.19 (0.08-0.31) | 0.000 | 0.03 (-0.01-0.07) | 0.170 | 11.1 |
| TAG 58:9 | -0.15 (-0.27--0.03) | 0.016 | -0.16 (-0.27--0.04) | 0.010 | 0.01 (-0.03-0.05) | 0.654 | NA |

β, 95% CI, *p*-value and PM were calculated from mediation analysis with adjustments for maternal age, pre-pregnancy BMI, nulliparity and family history of diabetes.

The value of proportion is indicated as NA (Not Applicable) when the direct and indirect effects are in opposite directions (VanderWeele, 2015).

Abbreviations: GDM, gestational diabetes mellitus; MAFLD, metabolic dysfunction-associated fatty liver disease; GCA, glycocholate; GCDCA, glycochenodeoxycholate; TCA, taurocholate; TDCA, taruodeoxycholate; TCDCA, taurochenodeoxycholate; FFA, free fatty acid; lysoPE, lyso phosphatidylethanolamine; PE, phosphatidylethanolamine; PI, phosphatidylinositol; LysoPC, lysophosphatidylcholine; SM, sphingomyelin; TAG, triacylglycerol.

**Supplementary Table S6. Baseline clinical, biochemical, and metabolic features of study populations in the second trimester.**

| Characteristics | non-GDM subjects | GDM subjects | *p*-value |
| --- | --- | --- | --- |
|  | (n=118) | (n=118) |  |
| ***Baseline characteristics in the second trimester*** | | | |
| Body mass index (kg/m^2^) | 23.8 (22.2 – 25.8) | 27.0 (23.6 – 29.8) | <0.001 |
| ***Laboratory results in the second trimester*** |  |  |  |
| Aspartate aminotransferase (IU/L) | 16.0 (13.2 – 21.0) | 13.0 (11.0 – 18.0) | 0.002 |
| Alanine aminotransferase (IU/L) | 10.0 (7.0 – 14.0) | 9.0 (6.5 – 13.5) | 0.661 |
| Total cholesterol (mg/dL) | 245.8 ± 80.8 | 241.5 ± 49.5 | 0.637 |
| High-density lipoprotein -cholesterol (mg/dL) | 80.0 ± 26.6 | 72.0 ± 17.6 | 0.010 |
| Low-density lipoprotein -cholesterol (mg/dL) | 122.3 ± 51.1 | 117.7 ± 40.5 | 0.475 |
| Triglycerides (mg/dL) | 189.5 (144.5 – 260.6) | 249.7 (195.6 – 320.8) | <0.001 |
| gamma-Glutamyl transferase (IU/L) | 9.0 (7.0 – 13.0) | 10.0 (8.0 – 17.0) | 0.057 |
| Glucose (mg/dL) | 84.0 (81.0 – 91.0) | 93.0 (85.5 – 99.0) | <0.001 |
| Data are presented as number (%), mean ± SD or median (interquantile range). | | | |
| *p*-value from chi-square, Student's t test or Mann-Whitney test as appropriate. | | | |

**Supplementary Figure S1. Flow chart of the statistical analysis.**


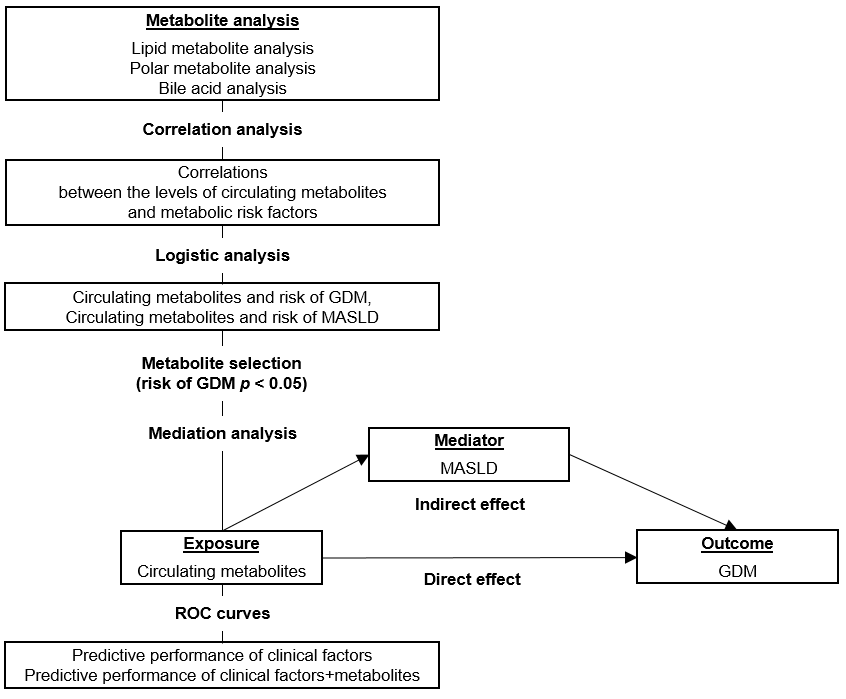


**Supplementary Figure S2. Pathway analysis.**


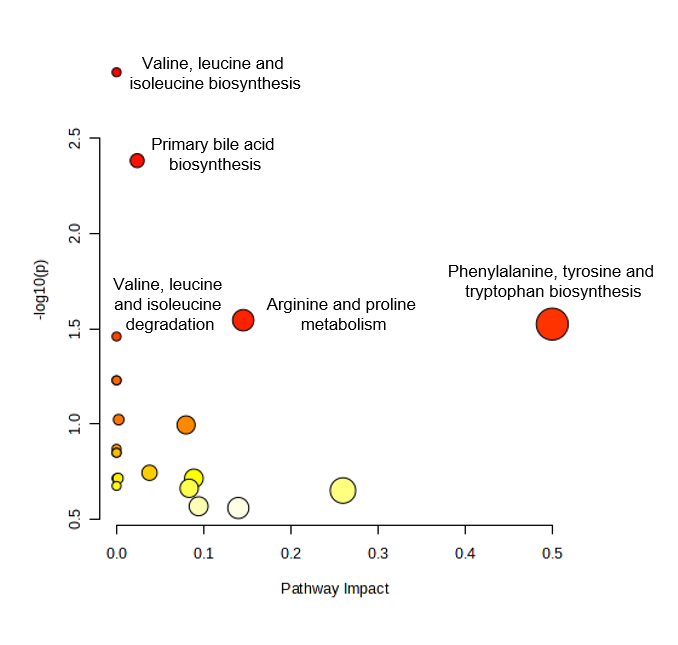

Supplement: Supplementary file 1 — Supplementary Material 1. [file 12933_2025_2645_MOESM1_ESM.docx]
